# On the possibility of tree-level leptogenesis from Kalb–Ramond torsion background

**DOI:** 10.1140/epjc/s10052-015-3731-z

**Published:** 2015-10-29

**Authors:** M. de Cesare, Nick E. Mavromatos, Sarben Sarkar

**Affiliations:** Theoretical Particle Physics and Cosmology Group, Department of Physics, King’s College London, Strand, London, WC2R 2LS UK; Theory Division, Physics Department, CERN, 1211 Geneva 23, CH Switzerland

## Abstract

In this work we consider a phenomenological model for leptogenesis in the context of a Standard Model Extension with an axial-like background coupling to fermions that violates both Lorentz and CPT symmetries. The latter is motivated by a background geometry of the early Universe involving a particular kind of torsion, arising from the Kalb–Ramond antisymmetric tensor field which appears in the gravitational multiplet of string theory, although we do not restrict ourselves to this framework. It is shown that leptogenesis can occur even at tree level and with only one generation of right-handed heavy Majorana neutrinos, due to ${ CP }$ and CPT violation introduced by the background geometry. Important issues for the model, including (a) its compatibility with a conventional-like cosmology and (b) current-era phenomenology (characterised by very stringent bounds on the allowed amount of torsion) are pointed out, and potential ways of resolving them, within the framework of string-theory models, are discussed.

## Motivation and summary

Baryogenesis represents a long-standing problem and is a very active research area in modern cosmology. A solution for baryogenesis would explain why the primordial Universe, which was dominated by radiation, evolved into the present matter dominated Universe. Many approaches, proposed in the literature, are reviewed in [1–7]. A standard measure of the abundance of baryons over that of antibaryons is defined by the ratio [8]

1 $$\begin{aligned} Y_{\Delta B}=\frac{n_{B}-n_{\bar{B}}}{n_{\gamma }}=(6.1\pm 0.3)\times 10^{-10} \end{aligned}$$

where $n_{B}$ is the number density of baryons, $n_{\bar{B}}$ is the number density of antibaryons and $n_{\gamma }$ is the density of photons (proportional to the entropy density *s*). This number was determined with accurate measurements of the CMB radiation by the experiments WMAP [9] and Planck [10]. However, there is no experimental evidence for primordial antimatter in the visible Universe. Similarly, the generation of an asymmetry between leptons and antileptons is known as leptogenesis. This is expected to be of the same order of magnitude as $Y_{\Delta B}$. If *B*, the net baryon number, is conserved in Nature, the matter asymmetry can only originate from an asymmetric initial condition $B\ne 0$. However, such an asymmetry would rapidly diminish during inflation, and extreme fine tuning of the initial condition would become necessary. This is highly unsatisfactory from a theoretical point of view. Consequently a mechanism for the dynamical generation of a baryon asymmetry is required. In the seminal paper [11–14], Sakharov identified three sufficient conditions that must be satisfied in order to produce a net baryon number.

1. The theory must allow for interactions that violate *B* conservation. These interactions must become effective at high-energy scales in order to guarantee the stability of the proton.
2. Both discrete symmetries *C* (charge conjugation) and ${ CP }$ (where *P* denotes parity) are violated. In fact *C* violation is not enough, as correlations between the spins of particles and antiparticles lead to identical cross sections for conjugated processes [15] when the theory is ${ CP }$ symmetric.
3. A departure from thermal equilibrium must occur: a *CPT* invariant theory (where *T* denotes time reversal) does not allow $\langle B\rangle \ne 0$ at thermal equilibrium.

A detailed review of Sakharov’s conditions in different baryogenesis models can be found in [3, 4]. The third Sakharov condition implicitly assumes that the underlying field theory is invariant under the discrete symmetry operator $\Theta \equiv CPT$. This assumption is usually valid due to the CPT theorem [16]: $\Theta$ is an invariance of local Lorentz invariant quantum field theories. $\Theta$ invariance is not always valid, for example (1) in models of spontaneous baryogenesis (see e.g. [17, 18]) and (2) through interactions with external fields [19] where the matter asymmetry is produced in equilibrium. Recently it was emphasised by Greenberg that CPT violation also implies Lorentz violation [20].

On closer inspection the Standard Model (SM) can be seen to satisfy the Sakharov conditions:

- At the classical level the Lagrangian of SM has global *U*(1) chiral symmetries, which lead to *B* conservation as well as *L* conservation for individual generations. At the quantum level, however, the currents of these global symmetries are anomalous [21–24]: $B+L$ is anomalous but $B-L$ is an exact symmetry of the quantum theory. Hence, in this framework, non-conservation of *L* implies non-conservation of *B* and leptogenesis implies baryogenesis. It was shown in [24] that processes which violate $B+L$ correspond to transitions between inequivalent gauge-field vacua, known as instantons [25, 26]. However, the probability of tunnelling is suppressed by an exponential factor governed by the potential barrier between vacua. The potential barrier can be overcome at high temperature [27]. This scenario, where leptogenesis implies baryogenesis, holds clearly within models in which SM can be embedded.
- Invariance with respect to *C* is manifestly broken in SM; invariance with respect to ${ CP }$ is broken by complex phases in the Yukawa couplings.
- The expansion of the Universe provides an out-of-equilibrium situation, A first order electroweak phase transition can also provide a non-equilibrium situation at the transition temperature. However, from the observed value of the Higgs mass, the transition is predicted to be continuous and, for this reason, it cannot lead to a significant departure from equilibrium [28, 29].

The SM, although it satisfies the Sakharov conditions, leads to a prediction for $Y_{\triangle B}$ which is several orders of magnitude smaller than its observed value [27]. Extra sources of ${ CP }$ violation beyond SM are needed. An important example of physics beyond the Standard Model is the oscillation [30] between different neutrino flavours; such oscillations require small non-zero neutrino mass differences which can be generated by the seesaw mechanism [31–35]. Three right-handed massive neutrinos are required in the seesaw mechanism [31–35] for the generation of the light (active) neutrino masses in the SM, which are much smaller than the masses of the right-handed-neutrino sector.

Fukugita and Yanagida [36, 37] used the extension of SM required by the seesaw mechanism to propose a model for leptogenesis: the lepton abundance is produced by the decay of heavy right-handed Majorana neutrinos [and so represents physics beyond the Standard Model (BSM)]. The difference in the branching ratios of the channels of production of leptons and antileptons is equal to the imaginary part of the interference term of tree-level and one-loop diagrams for the decay processes. For the interference to generate a non-zero ${ CP }$ violating phase, at least two generations of right-handed neutrinos are needed (see [36] and formulae therein).^1^ The model of Fukugita and Yanagida connects an explanation of leptogenesis to the seesaw mechanism. The model thus represents an economical extension of SM. However, the amount of ${ CP }$ violation required is hard to generate.

In fact, any theory of cosmology that does not explain baryogenesis can be considered as incomplete. The current explanations do not generate sufficient baryogenesis and so highlight the need for additional mechanisms for the generation of a baryon asymmetry, involving supersymmetry, extra-dimensional models etc. Gravitational effects are not incorporated in SM. Quantum gravity and the SM can, however, coexist within the framework of string theory.

Gravity in string theory [38] occurs as part of a massless multiplet (“gravitational”) comprising a spin-two massless field that is identified as the graviton, a scalar field, the dilaton, and a spin-one antisymmetric tensor field ${\mathfrak {B}}_{\mu \nu }=- {\mathfrak {B}}_{\nu \mu }$, the Kalb–Ramond (KR) field [39]; this will have interesting consequences. Although there are different types of string theory, the low-energy actions that emerge contain these massless fields. The effective action associated with the gravitational multiplet can be studied in lowest order in perturbation theory in the Regge slope $\alpha ^\prime = 1/M_s^2$ (with $M_s$ the string mass scale). The Regge slope is inversely proportional to the string tension. However, by working to all orders in $\alpha ^\prime$, it is possible to find a non-perturbative fixed point [40] which can have important consequences for leptogenesis: there is a torsion background which is constant in cosmic time and, in the presence of fermions, couples to the axial fermion current.

In this work we will use some ingredients of the gravitational sector of string theory [38] to propose a potentially new mechanism for baryogenesis via leptogenesis, but our considerations will not make detailed use of string-theory models, since they have some unresolved problems [41]. Such microscopic considerations will be the subject of future work. The geometry due to a background Kalb–Ramond field can lead to a Lorentz and ${ CP }$ violating interaction with fermions [42, 43] in theories with chiral anomalies. The corresponding field strength $H_{\mu \nu \rho } = \partial _{[\mu }\,{\mathfrak {B}}_{\nu \rho ]}$ (where $[\dots ]$ denotes antisymmetrisation of the respective indices) is proportional to the torsion of the background geometry, and is universally coupled to fermions via the affine connection. Such couplings (in specified backgrounds) belong to the class of interactions considered in the extension of the SM proposed in [44] and can be both Lorentz, ${ CP }$ and CPT violating. Moreover, in four space-time dimensions, the dual of the *H* field strength, $\epsilon ^{\mu \nu \rho \sigma } H_{\mu \nu \rho }$, may be represented as $\exp (2 \phi )\partial ^\sigma b(x)$, where *b*(*x*) is a pseudoscalar field—the ‘Kalb–Ramond’ (KR) axion.

**Fig. 1:**
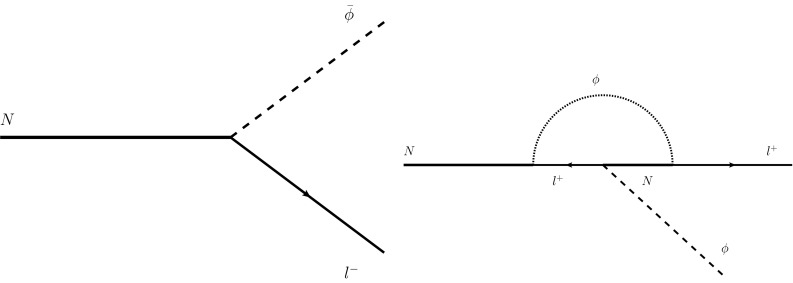
Tree- (*left*) and one-loop (*right*) decay amplitudes, corresponding to the Yukawa term that couples a right-handed neutrino to the standard model lepton sector. Analogous diagrams describe the decay in antileptons. Continuous *undirected lines* represent right-handed neutrinos, *lines* with an *arrow* are used to represent SM leptons, *whilst dashed lines* correspond to the SM Higgs. The *left* diagrams are understood to be evaluated in the presence of a KR background field. The *right* diagram is the standard result of [36], leading to Leptogenesis

At this point it should be noticed that the role of quantum fluctuations of the KR axion in theories with chiral anomalies has been previously considered in the context of the generation of chiral Majorana neutrino masses [45] beyond the seesaw mechanism,^2^ but not within the context of baryogenesis *per se*. We will argue in the present paper that the right-handed Majorana neutrinos, occurring in a Fukugita–Yanagida type leptogenesis model, will couple (along with all the other fermions) universally to a CPT-violating KR torsion background; this coupling will provide, through the decays of the Majorana neutrinos to SM sector in the presence of such backgrounds, new and universal sources of ${ CP }$ violation that could lead to leptogenesis, which can then transform to baryogenesis via SM $B-L$ conserving processes. If the KR torsion field had been large enough in the early Universe, we will show that sufficient leptogenesis can occur even with only one right-handed neutrino. A further feature is that the lepton asymmetry can be obtained even by only considering *tree-level* Feynman diagrams, unlike the standard leptogenesis scenarios, where ${ CP }$ violation arises at one-loop level. The diagrams represent the decays of a right-handed neutrino to a Higgs particle and a light left-handed lepton or the corresponding antilepton (because of Yukawa couplings). In order to study consistently such decays, the external lines of the pertinent Feynman diagrams must be treated non-perturbatively in the external field strength of the KR field background. When more generations of right-handed neutrinos are considered, there is an additive tree-level modification to the standard (one-loop) expression of the asymmetry derived in (cf. Fig. 1). On embedding our theory into the type-I seesaw models, we would naturally consider three right-handed neutrinos. However, if the masses of these heavy right-handed neutrinos are hierarchical, our considerations for leptogenesis would reduce to considering the lightest of these right-handed neutrinos.

The model should still be considered phenomenological. There remain important issues that should be addressed before the model can be considered to be realistic; these relate to the microscopic dynamics of the torsion field. In the current work we outline these problems. A particularly pressing issue, is to understand the reason for the virtual absence of such a leptogenesis-producing torsion field today: there are very stringent bounds imposed by a plethora of experimental tests of the SME.

In Sect. 2 we describe our phenomenological model: in addition to the SM fields, the model requires an extra right-handed Majorana neutrino, in the presence of an axial vector background for the fermions. The background violates both Lorentz and CPT symmetry. The model belongs to a class of models contained within the framework of the Standard Model Extension (SME). The right-handed neutrino couples to the SM sector via appropriate Yukawa couplings. These lead to decays of the right-handed neutrino to Higgs and active neutrinos, depicted in Fig. 1, which take place in the presence of a constant axial-background field in the observer’s frame. Such decays provide, already at *tree level*, extra sources for ${ CP }$ violation, which play an important rôle for leptogenesis, as discussed in Sect. 3. In Sect. 4 we discuss the possibility that a microscopic field-theoretic explanation for the constant axial background may be provided by the Kalb–Ramond *H*-torsion field in an early epoch of the Universe. Important issues for the torsion model, concerning cosmology and current phenomenology (e.g. the absence of any evidence for the existence of torsion today or its effects on the cosmic microwave background) are discussed in Sect. 5, where we also suggest potential ways of resolving some of them. Finally, conclusions and outlook are presented in Sect. 6. Some technical aspects of our work are given in several appendices, where we also discuss generic properties of field theories in space-time backgrounds with torsion, including dispersion relations, spinor chirality and helicity properties of Majorana spinors. These properties are required for understanding the precise way in which leptogenesis is realised in our model.

## Standard model extension with one right-handed neutrino in the presence of axial backgrounds

In this section we consider a *phenomenological* minimal extension of the Standard Model, with one right-handed massive (of mass *M*) Majorana neutrino field in the presence of constant axial backgrounds, $B_\mu$. The right-handed neutrino sector of such a model is described by2*N* is the Majorana field and $L_{k}$ is a lepton field, with *k* a generation index. The adjoint of the Higgs field is defined by the relation

3 $$\begin{aligned} \tilde{\phi }_i=\varepsilon _{ij}\phi _j \end{aligned}$$

We note that, since our primary motivation here is to identify the axial-background field with the totally antisymmetric part of a torsion background (cf. Appendix A), one should also consider the coupling of the axial field $B_\mu$ to all other fermions of the SM sector, $\psi _j$ (*j* = leptons, quarks) via a *universal* minimal prescription. Hence, the coupling with all fermionic species is the same: . Specifically, as we shall see in Sect. 4, the identification of the torsion background with a homogeneous and isotropic cosmological Kalb–Ramond field in a string-theory-inspired model will lead to axial backgrounds with non-trivial temporal components only

4 $$\begin{aligned} B_0 = \mathrm{const} \ne 0,\quad B_i = 0,\quad i=1,2,3. \end{aligned}$$

This will always be understood in what follows. In Appendix B we discuss properties of spinors coupled to such constant axial backgrounds (4), which prove very useful for a better understand of the associated leptogenesis scenarios studied in the next Sect. 3.

Since in SM the leptons have definite chirality, the Yukawa interactions can be rewritten as

5 $$\begin{aligned} \mathcal {L}_{YUK}=-Y_{k}\overline{L}_{k}\tilde{\phi }\left( \frac{1+\gamma ^{5}}{2}\right) N-Y_{k}^{*}\overline{N}\tilde{\phi }^{\dagger }\left( \frac{1-\gamma ^{5}}{2}\right) L_{k}. \end{aligned}$$

Using the properties of the charge conjugation matrix and the Majorana condition, it is again seen to be equivalent to

6 $$\begin{aligned} \mathcal {L}_{YUK}=-Y_{k}\overline{L}_{k}\tilde{\phi }\left( \frac{1+\gamma ^{5}}{2}\right) N-Y_{k}^{*}\overline{L}_{k}^{c}\tilde{\phi }^{\dagger }\left( \frac{1-\gamma ^{5}}{2}\right) N. \end{aligned}$$

It should be noted that the two hermitian conjugate terms in the Yukawa Lagrangian are also CPT conjugate. This is to be expected on the basis of the CPT theorem. In fact CPT violation is introduced only by interactions with the background field. In the absence of the background, the squared matrix elements obtained from tree-level diagrams for the two decays would be the same [46]. From the form of the interaction Lagrangian in Eq. (6), it is straightforward to obtain the Feynman rules for the diagrams giving the decay of the Majorana particle in the two distinct channels. It also allows us to use positive frequency spinors both for the incoming Majorana particle and for the outgoing leptons.

Let us now turn to the study of the *tree-level* decay processes of a Majorana right-handed neutrino into leptons and Higgs fields, depicted in Fig. 1. The total four-momentum is conserved in the decay. We use *p* to denote the four-momentum of the Majorana particle, *k* and *q* for the four-momentum of the Higgs and the outgoing (anti)lepton, respectively.

7 $$\begin{aligned}&E_{p,r} =E_{q,s}+E_{k}\end{aligned}$$

8 $$\begin{aligned}&\vec {p} =\vec {q}+\vec {k} \end{aligned}$$

Note that the energy of the fermions displays an explicit dependence on the helicity. Even assuming the decay products to be massless (which is legitimate, since leptons are actually massless in the unbroken electroweak phase and the Higgs mass parameter is expected to be much smaller compared to the other parameters with dimension of mass), kinematics has to be studied case by case, considering all the possible combinations of the external lines helicities. However, the analysis is much easier if one assumes that the right-handed neutrino is initially at rest. A discussion of the general case, along with a method to find approximate solutions, is given in Appendix C. In this case the following relations hold:

9 $$\begin{aligned} E_{p=0}=\sqrt{B_0^2+m^2}, \quad E_{k}=|\vec {k}|,\quad E_{q,s}=|B_0+\lambda _{s}|\vec {q}||. \end{aligned}$$

Momentum conservation also gives $|\vec {k}|=|\vec {q}|$.

We are then lead to consider two distinct cases, depending on the magnitude of the momentum:

*Case* (*a*) $$B_0+\lambda _{s}|\vec {q}|>0$$

From $s=2$ it follows that $m^2=0$; hence, for the decay of a massive particle, only $s=1$ is allowed and

10 $$\begin{aligned} |\vec {q}|=\frac{\Omega -B_0}{2}. \end{aligned}$$

In the last formula we introduced the quantity $\Omega$, defined as $\Omega =\sqrt{B_0^2+m^2}$.

*Case* (*b*) $$B_0+\lambda _{s}|\vec {q}|<0$$

From $s=1$ it follows that $m^2=0$. Therefore, for the decay of a massive particle, $s=2$ is the only allowed case and

11 $$\begin{aligned} |\vec {q}|=\frac{\Omega +B_0}{2}. \end{aligned}$$

We can finally turn to the calculation of the decay amplitudes, starting with the process $N\rightarrow l^{-}\overline{\phi }$. $U^{r}$ will denote the spinor wave function of the decaying particle and $u^s$ that of the lepton produced by the decay.

12 $$\begin{aligned} \mathcal {M}^{rs}&=-iY\overline{u}^s(q)\left( \frac{1+\gamma ^{5}}{2}\right) U^{r}(p)\nonumber \\&=-iY\xi _{s}^{'\dagger }\sqrt{q_{s}\cdot \sigma -B_0}\sqrt{p\cdot \overline{\sigma }+B_0}\,\xi _r\end{aligned}$$

13 $$\begin{aligned}&=iY\xi _{s}^{'\dagger }\xi _r\sqrt{E_{q,s}-|\vec {q}_{s}|\lambda _s-B_0}\sqrt{E_{p,r}+B_0+\lambda _{r}|\vec {p}|} . \end{aligned}$$

The notations $q_{s}$, $E_{s}$ are used to stress the dependence on the helicity of the four-momentum of the outgoing lepton, and similarly for the incoming particle. Helicity eigenstates corresponding to the outgoing lepton are primed. This is necessary since the momenta $\vec {p}$ and $\vec {q}$ are not parallel, which amounts in our formalism to the use of two distinct axes for the quantisation of the two spins. It is useful for what follows to calculate the scalar products of the two spinors appearing in (13). We choose the following helicity eigenstates for the decaying particle with spin along the third spatial direction:

14 $$\begin{aligned} \xi _{2}=\left( \begin{array}{c} 0\\ 1 \end{array} \right) , \quad \xi _{1}=\left( \begin{array}{c} 1\\ 0 \end{array} \right) . \end{aligned}$$

The corresponding helicity eigenstates, for the outgoing lepton emitted at angles $\theta , \phi$ (in spherical co-ordinates) are

15 $$\begin{aligned} \xi ^{'}_{2}=\left( \begin{array}{c} -e^{-i\phi }\sin \theta /2\\ \cos \theta /2\end{array}\right) , \quad \xi ^{'}_{1}=\left( \begin{array}{c} \cos \theta /2\\ e^{i\phi }\sin \theta /2\end{array}\right) . \end{aligned}$$

Since $E_{q,s}= \left|B_0+\lambda _{s}|\vec {q}_{s}|\right|$, in the amplitude we have to consider two cases, in the same way as we did for the kinematics.

*Case* (*a*) ($$B_0+\lambda _{s}|\vec {q}|>0$$)

In this case the first square root in (13) vanishes identically, leading to

16 $$\begin{aligned} \mathcal {M}^{rs}=0. \end{aligned}$$

*Case* (*b*) ($$B_0+\lambda _{s}|\vec {q}|<0$$)

17 $$\begin{aligned} \mathcal {M}^{rs}=-iY\xi _{s}^{'\dagger }\xi _r\sqrt{-2(B_0+\lambda _{s}|\vec {q}_s|)}\sqrt{E_{p,r}+B_0+\lambda _{r}|\vec {p}|}. \end{aligned}$$

In the case in which the right-handed neutrino is at rest, one knows from kinematics that only $s=2$ is allowed and

18 $$\begin{aligned} |\vec {q}|=\frac{\Omega +B_0}{2}. \end{aligned}$$

Therefore

19 $$\begin{aligned} \mathcal {M}^{r2}&=-iY\xi _{2}^{'\dagger }\xi _r\sqrt{-2\left( B_0-\frac{\Omega +B_0}{2}\right) }\sqrt{\Omega +B_0}\end{aligned}$$

20 $$\begin{aligned}&=-iY\xi _{2}^{'\dagger }\xi _r\sqrt{(\Omega -B_0)(\Omega +B_0)}=-iYm\xi _{2}^{'\dagger }\xi _r. \end{aligned}$$

It is important to stress that, as one can see from the last formula, when the spatial part of the total momentum vanishes the decay amplitude is just the standard one.

Calculations for the conjugate decay channel $N\rightarrow l^{+}\phi$ are completely analogous to the previous ones.

The transition amplitude is given by

21 $$\begin{aligned} \mathcal {M}^{rs}&=-iY^{*}\overline{u}^s(q)\left( \frac{1-\gamma ^{5}}{2}\right) U^{r}(p)\nonumber \\&=-iY^{*}\xi _{s}^\dagger \sqrt{q_{s}\cdot \overline{\sigma }+B_0}\sqrt{p\cdot \sigma -B_0}\,\xi _r. \end{aligned}$$

It is non-vanishing only in case (a), and it reduces to

22 $$\begin{aligned} \mathcal {M}^{rs}=-iY^{*}\xi _{s}^{'\dagger }\xi _r\sqrt{2(B_0+\lambda _{s}|\vec {q}_s|)}\sqrt{E_{p,r}-B_0-\lambda _{r}|\vec {p}|}. \end{aligned}$$

In the special case when $\vec {p}=0$ [remember that case (a) implies that only $s=1$ is allowed] this expression simplifies to

23 $$\begin{aligned} \mathcal {M}^{r1}=-iY^* m\xi _{1}^{'\dagger }\xi _r. \end{aligned}$$

We next proceed to discuss leptogenesis induced by a constant $B^0$ background (4), which, as discussed later, might be induced by *H*-torsion in string-cosmology [40].

## Axial-background-induced ${{ CP}}$ violation and leptogenesis

In this section we proceed to calculate the relevant quantities needed for an estimate of the lepton asymmetry induced by the axial background (4) within the framework of the Lagrangian (2).

For cosmological applications the thermally averaged decay rate [47] is relevant. This is given by

24 $$\begin{aligned}&\sum _{rs}\int \text{ d }\Pi _{N,r} \text{ d }\Pi _{l,s}\text{ d }\Pi _{\phi }f_N(p_N,r)(2\pi )^4\nonumber \\&\quad \delta ^{(4)}(p_{N,r}-p_{l,s}-p_\phi ) |M^{rs}(N\rightarrow l\phi )|^2, \end{aligned}$$

where we have used the following notation for the Lorentz-invariant measure:

25 $$\begin{aligned} \text{ d }\Pi _{X,r}=\frac{\text{ d }^3 p_{X}}{2E_{X,r}(2\pi )^3}. \end{aligned}$$

The momenta in the integrand depend explicitly on the spin of the incoming and outgoing particles; hence we separately evaluate each term in the sum (weighted by the respective distribution function). Evaluation of the integrals in the laboratory frame is preferred since going to the centre of mass frame, would introduce spatial components of $B_{\mu }$. Since we will be considering temperatures lower than the mass of the decaying particle it is a good approximation to consider the decaying particle to be at rest.

The zero temperature decay rate is obtained by integrating the squared amplitude multiplied by a kinematic factor. The latter results from the integration over momenta of the outgoing particles, enforcing energy-momentum conservation through a delta function. This leads to the integration measure

26 $$\begin{aligned}&\int \text{ d }\Pi _{l,s}\text{ d }\Pi _{\phi }(2\pi )^4\delta ^{(4)}(p_{N,r}-p_{l,s}-p_\phi )\nonumber \\&\quad =\int \frac{\text{ d }\varpi }{16\pi ^2}\frac{|\vec {k}|}{E_q+E_k\left( 1+\lambda \frac{B_0}{|q|}\right) \left( 1-\frac{|p|}{|k|}\cos \theta \right) } \end{aligned}$$

where $\text{ d }\varpi$ is the solid angle element, $\vec {k}$ is the momentum of the Higgs particle, $\vec {q}$ is the lepton momentum and $\lambda$ is the lepton helicity. When $\vec {p}=0$ the measure reduces to

27 $$\begin{aligned} \int \frac{\text{ d }\varpi }{16\pi ^2}\frac{|\vec {k}|}{\Omega +\lambda B_0}. \end{aligned}$$

We now make the simplifying assumption that the decaying particle is at rest, which is a good approximation for temperatures *T* satisfying $T\le m$. From four-momentum conservation it follows that

28 $$\begin{aligned} |\vec {k}|=|\vec {q}|=\frac{\Omega -\lambda B_0}{2}, \end{aligned}$$

where $\lambda$ is the helicity of the (anti)lepton produced in the decay and $\Omega =\sqrt{B_0^2+m^2}$ is the energy of the initial particle. It is worth noting that only the case $|\vec {q}|+\lambda B_0>0$ is allowed for the decay of a massive particle at rest, since the opposite sign in the inequality implies that $m^2=0$. (The instability of massless particles is a peculiar feature of Lorenz violating theories but is not relevant for our model.) In this special case one has for both channels $N\rightarrow l^{-}\overline{\phi }$ and $N\rightarrow l^{+}\phi$ that the squared matrix element, averaged over the initial spin, has the value $|Y|^2m^2/2$. This would seem to lead to a trivial result, implying that it is impossible to generate a lepton asymmetry with this mechanism when the temperature drops to a value comparable to the energy of the decaying particle. However, this conclusion is incorrect, since there is a non-trivial dependence of the kinematic factor on the background field. We have for the channel $N\rightarrow l^{-}\overline{\phi }$ the decay rate

29 $$\begin{aligned} \Gamma _1=\sum _k\frac{|Y_k|^2}{32\pi ^2}\frac{m^2}{\Omega }\frac{\Omega +B_0}{\Omega -B_0}, \end{aligned}$$

while, for the other channel, $N\rightarrow l^{+}\phi$, the decay rate is

30 $$\begin{aligned} \Gamma _2=\sum _k\frac{|Y_k|^2}{32\pi ^2}\frac{m^2}{\Omega }\frac{\Omega -B_0}{\Omega +B_0}. \end{aligned}$$

It is interesting to see that the decay rate of one process is obtained from the other upon flipping the sign of $B_0$. The total decay rate is

31 $$\begin{aligned} \Gamma =\Gamma _1+\Gamma _2=\sum _k\frac{|Y_k|^2}{16\pi ^2}\frac{\Omega ^2+B_0^2}{\Omega }. \end{aligned}$$

It is worthwhile observing that this mechanism can produce a lepton asymmetry even with *only one right-handed neutrino*, whereas the standard leptogenesis scenario [36] requires at least three generations. Moreover, the occurrence of leptogenesis here is just due to decay processes at tree level, since the required ${ CP }$ violation is introduced by the background field that enters in the external lines of Feynman diagrams.

The decay process goes out of equilibrium when the total decay rate drops below the expansion rate of the Universe, which is given by the Hubble constant [48]

32 $$\begin{aligned} \Gamma \simeq H=1, 66\,T^2 \mathcal {N}^{1/2} m_{P}^{-1}. \end{aligned}$$

Here $\mathcal {N}$ is the effective number of degrees of freedom of all elementary particles and $m_{P}$ is the Planck mass. From the last equation one can estimate the decoupling temperature $T_{D}$, in terms of the unknown parameters $\Omega$, |*Y*| and $B_0$, is

33 $$\begin{aligned} T_{D}\simeq 6.2\cdot 10^{-2} \frac{|Y|}{\mathcal {N}^{1/4}}\sqrt{\frac{m_{P}(\Omega ^2+B_0^2)}{\Omega }}. \end{aligned}$$

In order for the inverse decay to be suppressed by the Boltzmann factor, we have to impose the further requirement that $T_{D}\le \Omega$ when $\Gamma \simeq H$ (delayed decay mechanism [36, 48, 49]). From this condition one can determine a lower bound for the mass *m*. In fact we are lead to the following inequality:

34 $$\begin{aligned} z(\Omega ^2+B_0^2)\le \Omega ^3, \end{aligned}$$

where $z=3.8\cdot 10^{-3}\frac{m_{P}|Y|^2}{\mathcal {N}^{1/2}}$. If we require that the bound is satisfied for all values of $B_0$ we get

35 $$\begin{aligned} m^2\ge 1.09\,z^2. \end{aligned}$$

For us the Yukawa coupling *Y* is a free parameter. If we assume $|Y|\approx 10^{-5}$, $\mathcal {N}\approx 10^2$, we get an order of magnitude estimate for the lower bound of $\overline{m}\approx 100 \;\text{ TeV }$.

The lepton number density produced can then be estimated in the following way. By assumption all the neutrinos are at rest before the decay; hence with branching ratios of the decays are given by $r=\frac{\Gamma _1}{\Gamma }$ and $1-r$. The decay of a single neutrino produces the lepton number

36 $$\begin{aligned} \Delta L=r-(1-r)=2r-1=\frac{2\Omega B_0}{\Omega ^2+B_0^2}. \end{aligned}$$

Multiplying this quantity by the initial abundance of right-handed Majorana neutrinos at the temperature $T_D$ one gets an approximate estimate of the lepton number density. The density of the Majorana neutrinos is given by

37 $$\begin{aligned} n_{N}=\sum _{\lambda }\frac{1}{(2\pi )^3}\int \text{ d }^3 p\, f(p,\lambda ) \end{aligned}$$

where, as usual, $\beta$ is the inverse temperature, $\lambda$ denotes the helicity and $f(p,\lambda )$ is the corresponding Fermi–Dirac distribution function. At high temperatures this is well approximated by the Maxwell–Boltzmann function. Therefore we set

38 $$\begin{aligned} f(p,\lambda )=e^{-\beta \sqrt{m^2+(p+\lambda B_0)^2}}. \end{aligned}$$

We can rewrite (37) as

39 $$\begin{aligned} n_{N}&=\frac{1}{2\pi ^2}\sum _{\lambda }(I_{2}(-\lambda B_0,\beta ,m)-2\lambda B_0\,I_1(-\lambda B_0,\beta ,m)\nonumber \\&\quad +B_0^2\,I_0(-\lambda B_0,\beta ,m)). \end{aligned}$$

The functions in round braces are defined as follows:

40 $$\begin{aligned} I_{n}(a,\beta ,m)=\int _{a}^{\infty }\text{ d }p\; p^{n} e^{-\beta \sqrt{m^2+p^2}}. \end{aligned}$$

Retaining only terms that are at most linear in $B_0$ we see that the term proportional to $I_0$ drops and $I_1$ can be evaluated at the zeroth order in $B_0$.^3^ Moreover, we have

41 $$\begin{aligned}&I_1(0,\beta ,m)=\frac{1+\beta m}{\beta ^2}\,e^{-\beta m}\end{aligned}$$

42 $$\begin{aligned}&\quad \mathrm{and}\nonumber \\&I_2(-\lambda B_0,\beta ,m)=e^{-\beta m}\nonumber \\&\quad \times \left[ \frac{-\lambda B_0 \, m}{\beta }+\sqrt{\frac{\pi }{2}}\left( \frac{m}{\beta }\right) ^{\frac{3}{2}} \text{ Erfc }\left( -\lambda B_0\sqrt{\frac{\beta }{2m}}\right) \right] . \end{aligned}$$

The last formula, Eq. (42), is valid in the non-relativistic limit $\sqrt{m^{2}+p^2}\simeq m+\frac{p^2}{2m}$. The complementary error function is defined as the integral of the Gaussian function

43 $$\begin{aligned} \text{ Erfc }(z)=\frac{2}{\sqrt{\pi }}\int _{z}^{\infty }\text{ d }u\, e^{-u^2}. \end{aligned}$$

Since

44 $$\begin{aligned} \text{ Erfc }^{\prime }(z)=-\frac{2\, e^{-z^2}}{\sqrt{\pi }}, \end{aligned}$$

on expanding around $B_0=0$, $I_2$ reduces to,

45 $$\begin{aligned} I_2(-\lambda B_0,\beta ,m)=e^{-\beta m}\,\sqrt{\frac{\pi }{2}}\left( \frac{m}{\beta }\right) ^{\frac{3}{2}}+\mathcal {O}(B_0^2). \end{aligned}$$

It is now straightforward to see that, performing the sum over helicities in (39), one recovers the usual expression for the density of a non-relativistic species

46 $$\begin{aligned} n_{N}=e^{-\beta m}\,\left( \frac{m}{2\pi \beta }\right) ^{\frac{3}{2}}+\mathcal {O}(B_0^2). \end{aligned}$$

We assume that the right-handed neutrino density distribution follows closely the equilibrium distribution for $T\ge T_{D}$ and drops rapidly to zero at lower temperatures $T\le T_{D}$; furthermore the density of the sterile neutrino (normalised to the entropy density) is well approximated by a step function. Therefore we see, upon multiplying (36) by $n_{N}$, that the total lepton asymmetry produced in the full decay of the right-handed neutrino is given by

47 $$\begin{aligned} \Delta L^{TOT}=(2r-1)n_{N}=\frac{2\Omega B_0}{\Omega ^2+B_0^2}n_N \end{aligned}$$

The lepton asymmetry $\frac{\Delta L^{TOT}}{n_{\gamma }}$ is expected to be of the same order of magnitude of the baryon asymmetry (1). An order of magnitude estimate of the ratio $\frac{B_0}{m}$ can be found making use of the approximation $T_D\simeq m$ and retaining only first order terms in $\frac{B_0}{m}$.

Recalling that the photon number density is

48 $$\begin{aligned} n_\gamma \simeq \frac{2\zeta (3)}{\pi ^2}\,T^3\simeq 0.24\, T^3 \end{aligned}$$

and that

49 $$\begin{aligned} \frac{\Delta L}{n_\gamma }\simeq 10^{-10}, \end{aligned}$$

we estimate the ratio of the background field to the mass of the sterile neutrino to be

50 $$\begin{aligned} \frac{B_0}{m}\simeq 10^{-8}. \end{aligned}$$

The small value of this ratio also allows us to justify *a posteriori* the neglect of higher powers of $B_0$ in the formulae above. From the lower bound for the mass of 100 TeV found in (35), for the case where $Y = {\mathcal O}(10^{-5})$, we get an approximation for the smallest possible magnitude of the background field required in order for this mechanism to be effective $B_0\simeq 1\,\text{ MeV }$. If other mechanisms contributed to the lepton asymmetry in the Universe, or the Yukawa couplings assume smaller values, the minimum value of $B_0$ would be smaller than the one given here. Baryogenesis is then assumed to proceed via $B-L$ conserving processes in the SM sector of the model.

In order to get a more accurate estimate of $B_0$, the relevant Boltzmann equation will need to be studied. This requires a good approximation for the thermally averaged decay rates (24) of all the relevant processes and will be the subject of future research. Nevertheless, in Appendix D we construct the Boltzmann equation, with the simple purpose of demonstrating the differences induced by the background $B_0 \ne 0$.

## Field theory models with Kalb–Ramond torsion, chiral anomalies and constant axial backgrounds

In this section we suggest that the constant axial background $B_0$ of the previous sections may correspond to a totally antisymmetric Kalb–Ramond field that is a generic background in sigma model effective actions for string theory. For early eras, where gravitational effects are strong, it is interesting to study the behaviour of the gravitational multiplet which comprises the graviton, Kalb–Ramond field and dilaton. A detailed phenomenology which involves a string-theoretic construction encompassing a proper discussion of compactification, the emergence of the known particles and a consistent understanding of dark energy is beyond the scope of this work. Hence we will take a phenomenological approach whereby we will rely on calculations which have some validity in the early Universe to motivate the torsion background that we have introduced in our phenomenological model. As the Universe cools the parameter related to torsion in the model will be (phenomenologically) taken to suitably diminish. [There are many processes which can be relevant for the thermal history: fermions and gauge fields need to be considered, for example, in addition to the gravitational sector. An example of the evolution of torsion whose magnitude diminishes with time is given in [50] where tree-level string cosmology equations are solved. It is conceivable that, in the presence of fermions and gauge fields and higher order contributions (in the Regge slope, $\alpha ^\prime$) to the string-effective action, this behaviour may survive.] Detailed discussions on such aspects of the model are left for a future work.

Gravity is represented by the curvature of space-time and, in general relativity, the connection on space-time is taken to be torsion-free and metric-compatible. Hence it is uniquely determined to be the Levi-Civita connection (which is uniquely determined by the metric). More generally, in the tetrad formulation, we have two independent 1-forms,

51 $$\begin{aligned} e^{a}\equiv e^{a}_{\mu }\left( x \right) \mathrm{d}x^{\mu }, \quad \omega _{b}^{a} \equiv \omega _{b\mu }^{a}\left( x \right) \mathrm{d}x^{\mu }, \end{aligned}$$

with $e^{a}_{\mu }( x)$ the vielbein and $\omega _{b\mu }^{a}( x)$ the Lorentz (spin) connection,

We can introduce two related 2-forms: the curvature 2-form $R_{b}^{a}=\mathrm{d}\omega _{b}^{a}+\omega _{c}^{a}\wedge \omega _{b}^{c}$ and the torsion 2-form $T^{a}=\mathrm{d}e^{a}+\omega _{b}^{a}\wedge e^{b}$. If $T^{a}$ vanishes then $\omega _{b}^{a}$ and $e^{a}$ are not independent. From the principle of general covariance we know that we have an *SO*(3, 1) local invariance (manifest in the tetrad formalism). We can go from Lorentz and space-time indices via

52 $$\begin{aligned} \gamma ^{\mu }(x)&=e_{a}^{\mu }(x)\gamma ^{a},\quad g_{\mu \nu }(x)=\eta _{ab}e_{\mu }^{a}(x)e_{\nu }^{b}(x),\nonumber \\ e_{a}^{\mu }e_{b\mu }&=\eta _{ab} \end{aligned}$$

where $\gamma ^{\mu }(x)$ is the Dirac matrix, $g_{\mu \nu }(x)$ is the metric and $\eta _{ab}$ is the Minkowski metric.

The torsion [51–54], in terms of space-time indices, is a rank $$\begin{pmatrix} 1 \\ 2 \end{pmatrix}$$ tensor, antisymmetric in the lower indices $T^{\lambda }_{\mu \nu }=-T^{\lambda }_{\nu \mu }$. No clear evidence exists for a classical torsion field. Nevertheless, there has recently been some recent interest in torsion phenomenology (see for example [53–57]). As we will see, one good (theoretical) reason exists for the space-time connection having a non-vanishing torsion: the gravitational multiplet of string theory [58].

### Kalb–Ramond torsion and constant axial backgrounds

In the Einstein frame, to first order in the string amplitude, the bosonic part of the low-energy effective action (in four large target-space-time dimensions) is given by [58]

53 $$\begin{aligned} S&=\frac{1}{2\kappa ^2}\int \text{ d }^4x\;\sqrt{-g}\nonumber \\&\quad \times \big (R-2\partial ^{\mu }\phi \partial _{\mu }\phi -e^{-4\phi }H_{\lambda \mu \nu }H^{\lambda \mu \nu }- V(\phi )\big ), \end{aligned}$$

where $\frac{1}{\kappa ^2} \equiv \frac{M_{s}^2\Omega ^{c}}{8\pi } = \frac{1}{8\pi {G}}$, with *G* the four-dimensional (gravitational) constant, $M_{s}^2$ is the string mass scale, $\Omega ^{c}$ the (dimensionless) compactification volume in units of the Regge slope $\alpha ^\prime$ of the string and $V(\phi )$ is a dilaton potential. The field $H_{\lambda \mu \nu }$ appearing in the formula represents the field strength of the Kalb–Ramond field, $\mathfrak {B}_{\mu \nu }$, and is defined in analogy with the electromagnetic tensor $H_{\lambda \mu \nu }=\partial _{[\lambda }{\mathfrak {B}}_{\mu \nu ]}$. Square brackets denote antisymmetrisation over the enclosed indices. It is *important* to note that the sum of the graviton and the Kalb–Ramond terms in (53) can be rewritten as the scalar curvature $\overline{R}$ of a new connection [58], which is no longer symmetric in its last two indices, defined as

54 $$\begin{aligned} \overline{\Gamma }^{\lambda }_{\mu \nu }=\Gamma ^{\lambda }_{\mu \nu }+e^{-2\phi }H^{\lambda }_{\mu \nu } \ne \overline{\Gamma }^{\lambda }_{\nu \mu }. \end{aligned}$$

In the string-effective action this can be extended to include corrections [59–62] of higher order in $\alpha ^\prime$ . The antisymmetry of $H^\lambda _{\mu \nu }$ in its lower indices, shows the role of the field strength as a torsion tensor [51–54]. This suggests that this new connection (54) might be more fundamental than the Levi-Civita connection, and leads to different predictions whenever the Kalb–Ramond field is in a non-trivial configuration. We will adopt this point of view which has motivated the construction of our model. In [42, 43] a potential rôle of the *H* field for leptogenesis was emphasised. Here we will elaborate further on this issue.

The connection in (54) allows one to formulate the dynamics of matter fields minimally coupled to the gravitational and torsion background. The case of a Dirac spinor will be considered. (Non-minimal couplings of matter fields to torsion have also been considered in [53, 54].) The definition of the covariant derivative of a spinor requires the introduction of the tetrad $\{e_{a}^{\mu }\partial _{\mu }\}$.

In the local Lorentzian frame given by the tetrad, the action is the same as the flat one in minimal coupling, provided that ordinary derivatives are replaced by covariant ones $\partial _{a}\rightarrow \bar{\nabla }_{a}$ (with respect to the new connection). This is obtained by requiring that $\bar{\nabla }_{a}\psi$ transforms under a boost of the tetrad according to the spinor and vector indices it carries [51, 52]. The result that one finds in this way is the following:

55 $$\begin{aligned} \bar{\nabla }_{a}\psi =e_{a}^{\mu }\left( \partial _{\mu }+\frac{i}{2}\bar{\omega }_{b\mu c}\Sigma ^{bc}\right) \psi . \end{aligned}$$

In the formula above $\Sigma ^{ab}=\frac{i}{4}\left[ \gamma ^{a},\gamma ^{b}\right]$ is the generator of the Lorentz group representation on four-spinors, while $\bar{\omega }_{a\mu b}$ is the Ricci rotation coefficient, defined as

56 $$\begin{aligned} \bar{\omega }^{ab}_{\mu }=e^{a}_{\nu }\bar{\nabla }_{\mu }e^{b\nu }=e^{a}_{\nu }\left( \partial _{\mu }e^{b\nu }+\overline{\Gamma }^{\nu }_{\mu \lambda }e^{b\lambda }\right) . \end{aligned}$$

Therefore the action is

57 $$\begin{aligned} S_{\mathrm{Dirac}}&=\frac{1}{2} \int \text{ d }^4x\;\sqrt{-g}\, i\nonumber \\&\quad \times \left( \overline{\psi }\gamma ^{a}\bar{\nabla }_{a}\psi -\bar{\nabla }_{a}\overline{\psi }\gamma ^{a}\psi +2im\overline{\psi }\psi \right) . \end{aligned}$$

The second term is usually not written in flat space, as its contribution is equal to the first term plus a surface integral. However, the situation is different when space-time is not flat. In fact the second term is needed in order preserve unitarity, allowing for the cancellation of an anti-hermitian term involving the trace of the Ricci coefficients $\bar{\omega }^{a}_{\;ac}$.

The physical content of the new terms contained in the spin connection becomes clearer on rewriting the Dirac action (57) in the following way:

58 $$\begin{aligned} S_{\mathrm{Dirac}}&=\int \text{ d }^4x\;\sqrt{-g}\, \overline{\psi }\left( i\gamma ^{a}\partial _{a}+\widehat{B}_{d}\gamma ^5\gamma ^d-m\right) \psi \nonumber \\&\equiv S_{\mathrm{Dirac}}^\mathrm{free} + \int \mathrm{d}^4x \sqrt{-g}\, \widehat{B}_\mu J^{5\mu },\quad J^{5\mu }\equiv \bar{\psi }\gamma ^\mu \gamma ^5\psi \end{aligned}$$

where the axial vector $\widehat{B}^{d}$ is defined by

59 $$\begin{aligned} \widehat{B}^{d}=\frac{1}{4}\varepsilon ^{abcd}e_{a}^{\mu } \bar{\omega }_{b\mu c} = \frac{1}{4}\varepsilon ^{abcd}e_{a}^{\mu }\, e_{b\nu }\left( \partial _{\mu }e_c^{\nu } + e^{-2\phi }H^{\nu }_{\mu \lambda }\,e_c^{\lambda }\right) . \end{aligned}$$

In this last step we have used (56), (54) and the symmetry $\Gamma ^\lambda _{\mu \nu } = \Gamma ^\lambda _{\nu \mu }$ of the *torsion-free* Christoffel symbol,

In the special case of either flat (Minkowski) or Robertson–Walker space-times (which do not contain off-diagonal metric elements mixing temporal and spatial components), the axial vector $\widehat{B}^d$ is non-trivial and constitutes just the dual of the torsion tensor

60 $$\begin{aligned} \widehat{B}^d = -\frac{1}{4}\,\varepsilon ^{abcd}\,e^{-2\phi }\, H_{abc}. \end{aligned}$$

In four space-time dimensions

61 $$\begin{aligned} \widehat{B}^\mu = \partial ^\mu b, \end{aligned}$$

where *b*(*x*) is a pseudoscalar field [also termed the Kalb–Ramond (KR) axion field].

However, for a generic space-time there is also a derivative coupling of the spinor to the tetrad. Such an effective interaction with the gravitational background is not the only complication in dealing with spinors in curved space since the kinetic term involves the tetrad $\partial _{a}\equiv e_{a}^{\mu }\partial _{\mu }$ and is therefore dependent on the space-time point. The important point here is that Dirac (and similarly Majorana) spinors are naturally coupled to an axial field derived from the gravitational multiplet of string theory. As we have noted, this interaction leads to interesting cosmological consequences.

For a bosonic string theory (with four uncompactified dimensions) in non-trivial cosmological backgrounds, a world-sheet description has been provided by a sigma model that can be identified with a Wess–Zumino–Witten type conformal field theory [40]. This construction has led to exact solutions (valid to all orders in the Regge slope, $\alpha ^\prime$) for cosmological bosonic backgrounds with non-trivial metric, antisymmetric tensor and dilaton fields. Such solutions, in the Einstein frame, consist of (1) a Robertson–Walker metric with a scale factor $a(t) \sim t$ where *t* is the cosmic time, (2) a dilaton field $\phi$ that scales as $\phi (t) \sim -\mathrm{ln}a(t),$ and (3) a KR axion field scaling linearly with the cosmic time, $\overline{b} \propto t$ with $\overline{b}$ denoting the background value of *b* (cf. (61)). The resulting background axial vector $\overline{{\widehat{B}}}^d$ has only a non-trivial temporal component

62 $$\begin{aligned} \overline{b} \sim \mathrm{const}\, t , \quad \partial ^\mu \overline{b} \sim \epsilon ^{\mu \nu \rho \sigma }\, e^{-2\phi } \, H_{\nu \rho \sigma }, \end{aligned}$$

and

63 $$\begin{aligned} \overline{{\widehat{B}}}^0 \propto {\dot{\overline{b}}} = \mathrm{constant} \end{aligned}$$

in the Robertson–Walker frame.

### Torsion and fermions

Motivated by calculations of string amplitudes involving fermions, we will consider that the above gravitational background (with an asymmetric Christoffel symbol) characterises the minimal coupling of fermions (in lowest order in $\alpha ^\prime$). Lorentz invariance does not hold in the presence of the torsion background. If there are Lorentz-violating non-vanishing components of vacuum expectations of fermionic currents, the maintenance of rotational symmetries implies that only the temporal components of currents are allowed to condense. In the presence of fermions coupled to the torsion *H*-field as in (58), the four-dimensional low-energy effective action gives the following equations of motion for the graviton and antisymmetric tensor:

64 $$\begin{aligned}&\mathrm{graviton}:R_{\mu \nu } - \frac{1}{4} H_{\mu }^{\,\, \alpha \beta } H_{\nu \alpha \beta } = 8\pi G \nonumber \\&\quad \times \left( T^\psi _{\mu \nu } - \frac{1}{2} g_{\mu \nu } T^\psi + \text{ dilaton-derivative } \text{ terms } +\cdots \right) ,\nonumber \\&\mathrm{antisymmetric\,tensor}:\nonumber \\&\quad \times \partial ^\mu \Big (\sqrt{-g} e^{-2\phi }\, \big [ H_{\mu \nu \rho } - \epsilon _{\mu \nu \rho \sigma } J^{5\, \sigma } + \cdots \big ] \Big ) = 0, \end{aligned}$$

where $\ldots$ denotes higher order terms in $\alpha ^\prime$ in the gravitational part of the action, $T^\psi _{\mu \nu }$ is the stress-energy tensor of fermionic matter and $T^\psi = g^{\mu \nu } T_{\mu \nu }^\psi$. There is of course an equation of motion for the dilaton which provides additional constraints for the background. In order to simplify the analysis we will assume a constant dilaton below.

It is conceivable, as we shall argue, that in the presence of high temperature and densities of fermions (relevant for the early Universe), one may have (Lorentz-violating) *perturbative* fixed points corresponding to a constant *H*-torsion. Indeed, from the equation for the antisymmetric tensor field (assuming a constant dilaton) we observe that it can be solved upon using the pseudoscalar dual field $\overline{b}$ defined in (62):

65 $$\begin{aligned} \partial ^\mu \left( \sqrt{-g} \big [ \epsilon _{\mu \nu \rho \sigma } (\partial ^\sigma {\overline{b}} - \tilde{c} \, J^{5\, \sigma }) + \mathcal {O}\big ((\partial \overline{b})^3\big )\big ] \right) = 0, \end{aligned}$$

where $\tilde{c}$ is a constant of proportionality. From the above equations (in truncated form) it is clear that the fermion condensate can be a source of torsion, Hence the non-perturbative solution (63), derived in [40] is still qualitatively valid since, from (65), we have

66 $$\begin{aligned} \dot{\overline{b}} = {\tilde{c}}\langle J^{5}_0 \rangle =\tilde{c} \langle \psi ^\dagger _i \gamma ^5 \psi _i \rangle = \mathrm{constant} \ne 0 , \end{aligned}$$

where *i* runs over appropriate fermion species.

In [63] a calculation in strong coupling gauge theory supported the formation of axial vector fermion condensates. At weak gauge coupling the condensates cease to form. The gauge coupling and string coupling are related in string model building of the fundamental interactions. If the dilaton, rather than being a constant becomes more negative with time (as in the explicit solutions from bosonic string theory that we have earlier considered), the string coupling and gauge coupling decrease with time; so there will be a time (and a critical value of *g*, $g_{c}$) when the gauge coupling will be too weak to support a condensate. This is of course qualitative: currently we can only speculate that the value of $g_{c}$ is achieved in the era of leptogenesis. For a fundamental mechanism we would need to be quantitative but this has not been achieved.

There is another serious problem at the microscopic level related to the cosmological constant and the need for fine tuning, a generally unsolved problem. The time dependent pseudoscalar, with constant rate (66) induces a vacuum-energy term of the type of a positive cosmological constant once fluctuations around the background are allowed (see footnote). There are ways that negative contribution might arise to cancel the positive contribution but this remains speculation (and will be discussed elsewhere). Hence we do not have a microscopic derivation but rather a microscopic motivation for postulating our torsion background.

In summary, our backgrounds with torsion are non-thermal [43], and, characterise phenomenological string-inspired cosmologies; the effect of ${ CP }$-violation, induced by such backgrounds, on lepton asymmetry is the main point of this article. In late eras, when the axial-current condensate becomes vanishingly small, from (65) we find [upon ignoring (as subleading) the higher order $\mathcal {O}((\partial \overline{b})^3)$ terms] that the rate of change of the $\overline{b}$ field diminishes with the cosmic time as the cube of the scale factor

67 $$\begin{aligned} \dot{\overline{b}} \sim 1/a^{3}(t). \end{aligned}$$

We will make use of this result in Sect. 5, when we discuss the history of this Universe after the leptogenesis epoch.^4^ This integration in the path integral implies the incorporation of quantum fluctuations which lead to the appearance of repulsive four-fermion terms in the effective action. In our detailed analysis of leptogenesis we considered just the background torsion field; however, for an estimate of the energy budget of the Universe these fluctuations need to be included. The split of the $\widehat{B}$ field can be made explicit into the background $\overline{\widehat{B}}^\mu$ and quantum fluctuations, ${\widehat{\mathcal{B}}}^\mu$,

68 $$\begin{aligned} \widehat{B}^\mu = \overline{\widehat{B}}^\mu + {\widehat{\mathcal {B}}}^\mu \end{aligned}$$

where the background satisfies (63). The result for the relevant factor of the path integral after integration over the quantum fluctuations $\widehat{B}$ reads

69 $$\begin{aligned} \mathcal {Z} \propto \ \int D\psi \, D\overline{\psi }e^{i {\widetilde{S}}(\overline{\widehat{B}}) + i\int \mathrm{d}^4 x\,\sqrt{-g}\,\frac{3}{16}\,\kappa ^2 J_\mu ^5\,J^{5 \, \mu }} \end{aligned}$$

where $\widetilde{S} (\overline{\widehat{B}}) = S(\overline{\widehat{B}}) + S_{\mathrm{Dirac}}^\mathrm{free} + \int \mathrm{d}^4x \sqrt{-g}\, \overline{\widehat{B}}_\mu J^{5 \, \mu }$ is the action in the presence of the background torsion, given by the sum of (53), and (58). There is more related discussion in Appendix A.

## Open issues of *H*-torsion-induced leptogenesis and ways to resolve them

In a microscopic model an *H*-torsion-induced background $B_0$ of the above magnitude of 1 MeV would correspond to a *large positive* contribution to the cosmological constant, on account of the current-current term in the effective action (69); if uncancelled, this vacuum energy would modify the standard cosmology in the radiation dominated eras of the early Universe. There are possibilities whereby these fluctuations might be cancelled. Within the context of brane world quantum field theories there may be anti-de Sitter type contributions from the bulk. In such cases, there are negative vacuum-energy contributions to the (four-dimensional) brane vacuum. Such contributions may suppress the $B_0$-induced vacuum-energy contributions to acceptable levels so that the standard cosmology may apply (cf. Eq. (53)). In the context of axial condensates there is a phase transition whereby the gauge coupling is too weak for their formation [63].

Thus it is likely that, as the Universe cools down, the Lorentz-violating condensates, which require strong couplings and high densities in order to be formed, are destroyed via a suitable phase transition. At this transition the *B* field vanishes. In our scenario this temperature needs to be lower than the decoupling one for leptogenesis. The destruction of the fermion condensate at a temperature $T \simeq T_D$, would imply that the Kalb–Ramond-torsion-axion field *b* no longer varies linearly with the cosmic time but diminishes with the scale factor as in (67). If one assumes a cooling law for the Universe, of the form $a \sim T^{-1}$, then the $B^0$ torsion field would scale with the temperature as $T^3$, for $T \le T_D$ in this scenario. Taking that into account, the temperature of the Universe today (from the CMB measurements) is $T_\mathrm{CMB} = 2.725\,\mathrm{K} = 0.2348 ~\mathrm{meV}$, and assuming that the perturbative fixed point is dominant at temperatures of the order of $T \simeq T_D = 100$ TeV, we obtain a cooling law for the torsion $B_0$-field of the form

70 $$\begin{aligned} B_0 = c_0 \, T^3, \quad c_0 = \mathrm{1\,MeV}{(100\,\mathrm{TeV})^{-3}} = 10^{-42}\,\mathrm{meV}^{-2}. \end{aligned}$$

Thus, in such a scenario, the value of $B_0$ today would be of order

71 $$\begin{aligned} B_{0\, \mathrm{today}} = {\mathcal O}(10^{-44})\,\mathrm{meV}, \end{aligned}$$

which is much too small for any experimental detection. These and other considerations require further evaluation. For the purposes of this work we will just consider the model with a background field which in the present era, away from the leptogenesis era, is effectively absent. Hence detailed models will need to confirm that the corresponding temperature, at which such a destruction can happen, is much higher than the $\mathcal {O}(100)$ GeV temperature at which the lepton asymmetry is transferred to baryon asymmetries due the $B-L$ conserving processes in the SM sector of the model. This is an important issue for future study.

It should be noted that precision atomic experiments have placed stringent upper bounds on $B_0 \le {\mathcal O}(10^{-2})$ eV, within the context of experimental tests of the SME. see also: [44, 64, 65]. Another important issue is the effect of the constant antisymmetric torsion on the cosmic microwave background (CMB) radiation spectrum. If we assume that the CMB spectrum is largely due to fluctuations at the surface of last scattering, which occurs at redshifts $z = \mathcal {O}(10^3)$, then we observe that at the corresponding temperatures $T = T_{\mathrm{CMB}} \, (1 + z) \sim 10^{3}\, T_\mathrm{CMB}$, the value of

72 $$\begin{aligned} B_{0\, \mathrm{last\,scat}} \sim 10^9 \, B_{0 \, \mathrm today} = {\mathcal O}(10^{-35})~\mathrm{meV}, \end{aligned}$$

as follows from (70). This is very small to produce any observable effects in the CMB spectrum as can be seen from the following argument: one may consider higher-derivative terms in the effective action of photons propagating in a torsion background (as is the case in string-effective theories). One then encounters, among others, higher-covariant-derivative-with-torsion terms of the form appearing in the Lorentz-violating electrodynamics [66, 67], whose effects on cosmic microwave background radiation have been classified. Among those terms are terms of the form $T^{\alpha \lambda }_{\,\,\,\rho } F_{\alpha \nu }\, \partial _\lambda {\tilde{F}}^{\rho \nu }$ and $T^{\sigma \gamma }_{\,\,\,\delta } F_{\sigma \nu }\, \partial _\gamma {F}^{\delta \nu }$, where $T^{\mu \nu \rho }$ is the torsion field and $\tilde{F}_{\mu \nu }$ is the dual of the photon field strength $F_{\mu \nu }$. Such terms may be constrained by the mixing of electric (*E*-) and magnetic (*B*-) type polarisations of the CMB due to induced birefringence. With the strength of the Kalb–Ramond torsion in (72), the possible effects are well within the corresponding bounds.

In general, the association of the Kalb–Ramond torsion with a pseudoscalar axion-like field, implies constraints of the interactions of this field with electromagnetic fields through the anomaly Eq. (A12). In our model, the coupling of this interaction turns out to be the gravitational coupling (A11), and thus such effects are very small, compatible with the current phenomenology.

## Conclusions and outlook

In this work we have given a concrete phenomenological model for leptogenesis based on our earlier work [42, 43] on the rôle of string-inspired Kalb–Ramond torsion in leptogenesis. Unlike the case of torsionless Riemannian manifolds, the presence of torsion associated with the totally antisymmetric Kalb–Ramond field strength, can imply for certain backgrounds, a lepton-number asymmetry in the early Universe: a consequence of different decay rates of heavy right-handed neutrinos at early epochs into leptons and antileptons. This difference is induced exclusively by the torsion, which can be constant in the (Einstein) Robertson–Walker frame. Our approach exploits a tree-level ${ CP }$ violating asymmetry, in contrast to the standard approach to leptogenesis where the asymmetry appears first at one-loop level.

We give only an order of magnitude estimate (in flat space-times) of the induced asymmetry. More detailed estimates, obtained from solving the Boltzmann equations in the presence of torsion, need to be done in future. Nevertheless, for completeness, in the current article we have also sketched the modifications induced by the torsion field in the *collisionless* Boltzmann equation and derived the associated particle distribution function, which was found to be well behaved for non-zero values of the temporal component of the axial vector.

Our simplified model for leptogenesis involves a single flavour of a heavy Majorana neutrino and the Yukawa coupling *Y* that couples it to the standard model lepton sector. It is possible to consider more complicated models in which there is a mixing between the fluctuations of the torsion pseudoscalar field and the usual axion fields. Yukawa interactions of these fields with the left-handed neutrino sector can produce dynamical generation of Majorana neutrino masses via higher-loop anomalous graphs [45]. Embedding of such a scenario in detailed microscopic string models may lead to restrictions on the allowed constant values of the torsion.

We would also like to comment that our model is discussed within the framework of thermal equilibrium leptogenesis. As discussed in Appendix B, the coupling of fermions to the axial field $B_{\mu }$ induces different dispersion relations for states with opposite helicity. The density of a given particle species is indeed given by

73 $$\begin{aligned} n=\frac{g}{(2\pi )^3}\int \text{ d }^3 p f(p), \end{aligned}$$

where *g* is the number of degrees of freedom and *f*(*p*) the probability distribution function in momentum space. For fermions this is a Fermi–Dirac function

74 $$\begin{aligned} f(p)=\frac{1}{\exp {\frac{E(p)}{kT}}+1}. \end{aligned}$$

When Lorentz-violating interactions give leptons and antileptons different energies for corresponding values of the momentum and helicity quantum numbers (analogous to the discussion of equilibrium baryogenesis in [44]), then the lepton asymmetry can be calculated as

75 $$\begin{aligned} n_{L}-n_{\overline{L}}=\frac{g}{(2\pi )^3}\int \text{ d }^3 p \; (f_{L}(p)-f_{\overline{L}}(p)). \end{aligned}$$

In [44] interactions are considered that lead to a uniform shift of the energy levels. The shift can be interpreted as a chemical potential that happens to be different for particles and antiparticles due to CPT violation. However, the present case is different, since particles and antiparticles with the same helicity have the same dispersion relation and hence the same density. There is a difference in density just between positive and negative helicity states, regardless of the fact that they belong to the same or to different particle species. For this reason there is no lepton asymmetry at equilibrium that can be justified on the basis of the CPT-violating interaction in the Lagrangian (58).

However, there is an asymmetry at equilibrium between right-handed neutrinos and anti-neutrinos that can be interpreted as a particle-antiparticle asymmetry. The right-handed neutrino is a weak isospin singlet, and is therefore allowed to have a Majorana mass. Since a Majorana particle is C-conjugated, the only way to distinguish a neutrino from its antineutrino is via ${ CP }$ conjugation, or equivalently by the helicity. Therefore the asymmetry between opposite helicity right-handed neutrino states amounts to an asymmetry between the density of neutrinos and antineutrinos. However, this cannot be interpreted in terms of a lepton asymmetry, since the right-handed Majorana neutrino has no definite lepton number. The way this asymmetry might contribute to leptogenesis is only through the decay of the right-handed neutrino, i.e. when this neutrino states are converted to other states having a definite lepton number. This constitutes the mechanism considered in our paper.

There are several open issues associated with the torsion-induced leptogenesis scenario presented here. One of them concerns the order of the vacuum energy during the leptogenesis era, which could affect the cosmological evolution in a serious way. Such vacuum energies should be small. In principle, this can be guaranteed in brane Universe models by a cancellation of the (large) kinetic energy of the torsion KR field on the brane (required for leptogenesis) by the anti-de Sitter (negative) contributions to the brane world vacuum energy due to structures in the bulk space. Detailed, and phenomenologically realistic, string/brane models, where such cancellations are demonstrated explicitly, are left for future investigations. Another important issue is that any trace of the torsion field today has to be very small in order to avoid violation of the very stringent experimental bounds. This implies the necessity for mechanisms in the early Universe by which the torsion disappears or it is diminished significantly in the current era. A possible mechanism is the phase transitions that destroys the axial current fermion condensates responsible for non-zero torsion [63]; this possibility awaits detailed confirmation within specific models.

In general, though, despite its problems, we believe that the novel mechanism for geometry-induced leptogenesis we propose in this work has its merits and deserves further studies in the future.

## Appendix A: Fermions and (quantum) torsion: generic properties

In this section we make connection with the well-known [53, 54] field-theoretic result that a theory of fermions in a space-time with torsion (Einstein–Cartan theory) results in a four-fermion interaction after integrating out torsion in a path integral. This is easily understood by the fact that the torsion is a non-propagating field in the Einstein–Cartan theory, where the gravitational field dynamics is described only by a generalised scalar curvature term coupled to Dirac fermions (which may or may not be charged)

A1 $$\begin{aligned} S_\mathrm{EC}&= \frac{1}{8\pi {G}} \, \int \mathrm{d}^4 x \sqrt{-g}\left( \bar{R}(\bar{\omega }) - S_\psi \right) ,\nonumber \\ S_\psi&= \frac{i}{2} \int \mathrm{d}^4 x \sqrt{-g} \left( \bar{\psi } \gamma ^\mu \bar{\mathcal {D}}_\mu \psi - (\bar{\mathcal {D}}_\mu \bar{\psi } ) \gamma ^\mu \psi \right) \end{aligned}$$

where $\bar{\mathcal {D}}_\mu = \bar{\nabla }_\mu - i e A_\mu$, is the covariant derivative of with *e* the fermion charge and $A_\mu$ an electromagnetic field. The overline above the covariant derivative, i.e. $\bar{\nabla }_\mu$, denotes the presence of torsion, which is introduced through the modified spin connection $\bar{\omega }$ :

A2 $$\begin{aligned} \bar{\omega }_{a \mu b } = \omega _{a \mu b } + K_{a \mu b}, \end{aligned}$$

where $K_{a \mu b}$ is the contorsion tensor (as usual Greek letters denote components in the coordinate basis, while Latin indices refer to the tetrad basis). The contorsion tensor is related to the torsion two-form $\mathbf {T} = \mathbf {de} + \varvec{\bar{\omega }} \wedge \mathbf {e}$ via [51–54, 62]:

A3 $$\begin{aligned} K^{\lambda }_{\;\mu \nu } = \frac{1}{2} \Big ( {T}^{\lambda }_{\;\mu \nu } + {T}_{\mu \;\nu }^{\;\lambda } + {T}_{\nu \;\mu }^{\;\lambda } \Big ). \end{aligned}$$

Apart from the standard terms in manifolds without torsion, the presence of torsion in the covariant derivative in the Dirac-like action (A1) leads, to an additional term involving the total axial current (the sum runs over all fermion species *k*) $J^\mu _5 \equiv \sum _{k}\bar{\psi }_{k} \, \gamma ^\mu \, \gamma ^5 \, \psi _{k}$:

A4 $$\begin{aligned} S_\psi \ni - \frac{3}{4} \int {\mathrm{d}}^4 \sqrt{-g} \, S_\mu \sum _{k}\bar{\psi }_{k} \, \gamma ^\mu \, \gamma ^5 \, \psi _{k} = - \frac{3}{4} \int \mathbf {S} \wedge {}^\star \! \mathbf {J}^5 \end{aligned}$$

where $\mathbf {S} = {}^\star \! \mathbf {T}$ is the dual of **T**: $S_d = \frac{1}{3!} \epsilon ^{abc}_{\quad d} T_{abc}$. In (A4), and in what follows, we adopt for notational convenience the language of differential forms to describe the effective action of fermions in a curved space-time with torsion.

We next remark that the torsion tensor can be decomposed into its irreducible parts [53, 54], of which $S_d$ is the pseudoscalar axial vector:

A5 $$\begin{aligned} T_{\mu \nu \rho } = \frac{1}{3} \big (T_\nu g_{\mu \rho } - T_\rho g_{\mu \nu } \big ) - \frac{1}{3!} \epsilon _{\mu \nu \rho \sigma } \, S^\sigma + q_{\mu \nu \rho }, \end{aligned}$$

with $\epsilon _{\mu \nu \rho \sigma } q^{\nu \rho \sigma } = q^\nu _{\,\rho \nu } = 0$. This implies that the contorsion tensor undergoes the following decomposition:

A6 $$\begin{aligned} K_{abc} = \frac{1}{2} \epsilon _{abcd} S^d + {\widehat{K}}_{abc} \end{aligned}$$

where $\widehat{K}$ includes the trace vector $T_\mu$ and the tensor $q_{\mu \nu \rho }$ parts of the torsion tensor.

The gravitational part of the action can then be written as:

A7 $$\begin{aligned} S_G =\frac{1}{2\kappa ^2} \, \int \mathrm{d}^4 x \sqrt{-g} (R + \widehat{\Delta } ) + \frac{3}{4\kappa ^2} \int \mathbf {S} \wedge {}^\star \mathbf {S}, \end{aligned}$$

where $\widehat{\Delta }= {\widehat{K}}^\lambda _{\, \, \mu \nu } {\widehat{K}}^{\nu \mu }_{\quad \lambda } - {\widehat{K}}^{\mu \nu }_{\quad \nu } \, {\widehat{K}}^{\quad \lambda }_{\mu \lambda }$, with the hatted notation defined in (A6).

In a *quantum* setting, where one integrates over all fields, the torsion terms appear as non-propagating fields and thus they can be integrated out exactly. The authors of [62] have observed though that the classical equations of motion identify the axial-pseudovector torsion field $S_\mu$ with the axial current, since the torsion equation yields

A8 $$\begin{aligned} K_{\mu a b} = - \frac{1}{4} e^c_\mu \epsilon _{a b c d} \bar{\psi } \gamma _5 \gamma ^d \psi \, \end{aligned}$$

From this it follows that $\mathbf {d}\,{}^\star \!\mathbf {S} = 0$, leading to a conserved “torsion charge” $Q = \int {}^\star \! \mathbf {S}$. To maintain this conservation in quantum theory, one has to postulate

A9 $$\begin{aligned} \mathbf {d}\,{}^\star \!\mathbf {S} = 0, \end{aligned}$$

at the *quantum* level, which can be achieved by the addition of judicious counter terms (see [62]). This constraint, in a path-integral formulation of quantum gravity, is then implemented via a delta function constraint, $\delta (\mathbf {d}\,{}^\star \! \mathbf {S})$, and the latter via the well-known trick of introducing a Lagrange multiplier field $\Phi (x) \equiv (3/2\kappa ^2)^{1/2} b(x)$. Hence, the relevant torsion part of the quantum-gravity path integral would include a factor

A10 $$\begin{aligned} {\mathcal Z}&\propto \int D \mathbf {S} \, D b \, \exp \left[ i \int \frac{3}{4\kappa ^2} \mathbf {S} \wedge {}^\star \! \mathbf {S} - \frac{3}{4} \mathbf {S} \wedge {}^\star \! \mathbf {J}^5 \right. \nonumber \\&\qquad \qquad \qquad \qquad \qquad \left. + \left( \frac{3}{2\kappa ^2}\right) ^{1/2} \, b \, \mathbf {d} {}^\star \! \mathbf {S} \right] \nonumber \\&= \int D b \, \exp \left[ -i \int \frac{1}{2} \mathbf {d} b\wedge {}^\star \! \mathbf {d} b + \frac{1}{f_b}\mathbf {d}b \wedge {}^\star \! \mathbf {J}^5 \right. \nonumber \\&\qquad \qquad \qquad \qquad \left. + \frac{1}{2f_b^2} \mathbf {J}^5\wedge {}^\star \! \mathbf {J}^5 \right] , \end{aligned}$$

where

A11 $$\begin{aligned} f_b = (3\kappa ^2/8)^{-1/2} = \frac{M_P}{\sqrt{3\pi }}, \quad J^{5 \, \mu } = \sum _{k}\bar{\psi }_{k} \, \gamma ^\mu \, \gamma ^5 \, \psi _{k}, \end{aligned}$$

and the non-propagating $\mathbf {S}$ field has been integrated out. The reader should notice that, as a result of this integration, the corresponding *effective* field theory contains a *non-renormalizable* repulsive four-fermion axial current-current interaction.

We may partially integrate the second term in the exponent on the right-hand-side of (A10) and take into account the well-known field-theoretic result that in QED the axial current is not conserved at the quantum level, due to anomalies, but its divergence is obtained by the one-loop result:

A12 $$\begin{aligned} \nabla _\mu J^{5 \, \mu }&= \frac{e^2}{8\pi ^2} {F}^{\mu \nu } \widetilde{F}_{\mu \nu } - \frac{1}{192\pi ^2} {R}^{\mu \nu \rho \sigma } \widetilde{R}_{\mu \nu \rho \sigma } \equiv G(A, \omega ). \end{aligned}$$

Observe that in (A12) the torsion-free spin connection has been used. This can be achieved by the addition of proper counter terms in the action [62], which can convert the anomaly from the initial $G(\mathbf {A}, \bar{\omega })$ to $G(\mathbf {A}, \omega )$. Using (A12) in (A10) one can then obtain for the effective torsion action in QED

A13 $$\begin{aligned}&\int D b\ \exp \left[ - i \int \frac{1}{2} \mathbf {d} b\wedge {}^\star \mathbf {d} b - \frac{1}{f_b} b G(A, \omega )\right. \nonumber \\&\qquad \qquad \qquad \left. + \frac{1}{2f_b^2} \mathbf {J}^5 \wedge ^\star \!\mathbf {J}^5 \right] . \end{aligned}$$

Thus, we observe that the torsion lead to repulsive four-fermion interactions involving the axial current. Crucial to the above derivation was, however, the postulation of the conservation of the torsion charge at the quantum level, as expressed by the constraint $\mathbf {d} {}^\star \! \mathbf {S} = 0$. The resulting axion field has originated from the Lagrange multiplier field implementing this constraint. In the subsequent section we consider the cosmological implications of this result. The reader should notice that the form of the action (A13) is like the one that can be derived from string-theory considerations with the Kalb–Ramond field as torsion (69), thereby establishing the close parallel of the two approaches.

The previous considerations imply that, in a four-dimensional space-time with a non-vanishing KR torsion, the effective field theory of *N* species of massive fermions $\psi _i$, $i=1, \dots N$, will be necessarily interacting via contaxt axial-current–current four-fermion terms, given by the Lagrangian [53, 54] (69):

A14

where $e^\mu _a$ are the vierbeins, *e* is the vierbein determinant, the suffix ;  denotes the covariant derivative with respect to the torsion-free space-time connection, $\widetilde{B}_\mu \equiv \overline{\widehat{B}}_\mu$, is the axial background, and the $\dots$ denote higher order terms that are present in the string-inspired theory. Summation over the fermion flavour indices $j, \ell = 1, \dots N$ is understood. In our approach the non-renormalizable four-fermion interactions arise on integrating out the torsion field (as noted in (69)).

We shall consider both Dirac and Majorana spinors in the framework of the interacting theory (A14). In order to discuss the effects of torsion on particle-antiparticle induced asymmetry, we consider the equations of motion for the spinors (and their charge conjugates) that follow from the Lagrangian (A14). The four-fermion interaction term will induce a cubic term in the equations of motion for the fermions. Such non-linear equations first appeared in the 1970s work of Hehl and Datta [68] and are now known eponymously. Under the assumption of formation of a (Lorentz-violating) fermionic condensate of the axial current, the Hehl–Datta equation is linearised.

It was recently argued [55] that Dirac fermions may lead to C- and CPT-violating differences between the fermion-antifermion populations in the finite temperature environment of the early Universe. However, the author did not consider that, after integrating out the torsion, the effective four-fermions interaction is such that all the fermionic species must contribute to one and the same condensate. This will turn out to be important for our purposes. We will consider the non-linear equations stemming from (A14) for both the Dirac spinor and the charge-conjugate spinor $\psi ^c = C{\overline{\psi }}^T$, where *T* indicates matrix transposition, and C is the (unitary) charge conjugation matrix, $C=i \gamma ^2 \gamma ^0$, in standard notation (no sum over *j* index):

A15

where, to obtain the second line, we used the Dirac equation obtained from (A14) for the Dirac conjugate spinor, took the transpose “*T*”, and acted upon from the left with the C-conjugation operator, using $-C\, \gamma _\mu ^T \,\text{ C }^{-1} = - \gamma _\mu$ and $C\, \gamma ^{5\, T}\, \text{ C }^{-1} = \gamma ^5$. We also used^5^

A16 $$\begin{aligned} \overline{\psi }_\ell ^c \gamma ^5 \, \gamma _a \, \psi _\ell ^c = - \overline{\psi }_\ell \, \gamma ^5 \, \gamma _a \, \psi _\ell . \end{aligned}$$

In a Hartree–Fock approximation, we may linearise the equations (A15) by replacing the fermion bilinear in the non-linear terms with its vacuum expectation value $\mathcal {F}_{\mu }\equiv \langle \overline{\psi }\gamma ^{5}\gamma _{\mu }\psi \rangle$. For isotropic situations, as is the case we are interested in, only its temporal component is non-trivial, and denotes the appropriate fermion chiral densities (summed up over all species).

A17 $$\begin{aligned} {\mathcal F}_0 = \langle \psi _\ell ^\dagger \gamma ^5 \psi _\ell \rangle \equiv \rho _R - \rho _L, \quad {\mathcal F}_i = 0. \end{aligned}$$

The linearised Hehl–Datta equations (A15) become (assuming also only a $\widetilde{B}^0 \ne 0$ component, for concreteness, as appropriate for our string-inspired case (62), which we restrict ourselves to here)

A18 $$\begin{aligned} i \, e^\mu _{\, a} \gamma ^a \psi _{j;\, \mu } - m \psi _j - \left( \widetilde{B}_0 + \frac{3\, \kappa ^2}{8} \, {\mathcal F}_0 \right) \, \gamma ^0 \, \gamma ^5 \, \psi _j&= 0 \nonumber \\ i\, e^\mu _{\, a} \gamma ^a \psi ^c_{j;\, \mu } - m \psi _j^c - \left( \widetilde{B}_0 + \frac{3\, \kappa ^2}{8} \, \mathcal {F}_0 \right) \, \gamma ^0\, \gamma ^5\, \psi _j^c&= 0. \end{aligned}$$

In [55], the difference in sign of the cubic fermion terms in (A15), between the fermions and their Dirac conjugate, have been interpeted as leading to different dispersion relations for constant background torsion and through this baryogenesis in the early Universe. Unfortunately we do not agree with this interpretation. In terms of (A16) we observe that in a Hartree–Fock approximation the isotropic condensate of the chiral current (interpreted as torsion) couples to matter and antimatter *with the same sign*, and hence there is no induced difference in the corresponding dispersion relations.

Moreover, a Majorana spinor, of interest to us in the context of the model (2), can be defined as $\nu = \psi + \psi ^c$ and is, by construction, a mass eigenstate, satisfying the Majorana condition $\nu ^c = \nu$, entailing that a Majorana fermion is its own antiparticle and is chargeless. From this condition, we observe that Majorana spinors do not contribute to the condensate ${\mathcal F}_0$. Of course the torsion mixes the Majorana neutrinos with all other fermion species, and thus non trivial backgrounds ${\mathcal F}_0$ are experienced in general by Majorana fermions in such space times with torsion. It follows directly from the definition that a Majorana spinor satisfies the same Eq. (A18). We observe that the quantities ${\mathcal F}_0$ are in general temperature dependent.

The above discussion demonstrates, therefore, that the constant axial background (4) of the phenomenological model (2) may be traced back to a microscopic string-theory-inspired model with Kalb–Ramond cosmological torsion, with non-trivial temporal components from the point of view of a cosmological observer given by the combination

A19 $$\begin{aligned} B_0 \equiv \left( \widetilde{B}_{0} + \frac{3\, \kappa ^2}{8} \, \mathcal {F}_{0} \right) ,\quad B_i = 0, \end{aligned}$$

where only temporal components of $B_\mu$ are non-zero, in accordance with previous considerations (A17), (63), (66). In (A19), $\widetilde{B}_{0}$ includes contributions to constant *H*-torsion background pertaining to the non-perturbative (exact in $\alpha ^\prime$) analysis of [40]. The considerations in Sect. 3 therefore, imply that in the presence of such constant torsion backgrounds, leptogenesis in the fermion sector can occur, yielding phenomenologically acceptable values for the lepton asymmetry if $B_0$ of order 1 MeV at 100 TeV temperatures are attained. The model, however, is far from being complete, when the background field $B_0$ is viewed as a string inspired dynamical torsion.

## Appendix B: Some properties of spinors coupled to an axial-background field

Let one consider the effective theory of spinors on flat space-time^6^ given by (58)

B1 $$\begin{aligned} S_{Dirac}=\int \text{ d }^4x\;\sqrt{-g}\, \overline{\psi }\left( i\gamma ^{\mu }\partial _{\mu }+B_{\mu }\gamma ^5\gamma ^\mu -m\right) \psi \end{aligned}$$

To avoid cumbersome notation $B_{\mu }$ here will denote the most general background torsion, including finite density condensates of the axial current, which violate Lorentz symmetry, in the finite density and temperature of the early Universe,

B2 $$\begin{aligned} B_\mu \equiv \left( \widetilde{B}_{\mu } + \frac{3\, \kappa ^2}{8} \, \mathcal {F}_{\mu } \right) , \end{aligned}$$

where only temporal components of $B_\mu$ are assumed to be non-zero.

This action belongs to the class of theories termed SME and considered in [44]. When the torsion is *constant* throughout space-time the interaction term leads to the spontaneous breakdown of Lorentz symmetry. The interaction terms with coefficients $B_\mu$ are known to be *both* Lorentz and CPT violating in that case.

### Dispersion relations of fermions in a constant axial background

When $B_{\mu }$ is constant, it makes sense to look for plane-wave solutions of the equations of motion. The dispersion relations thereby obtained are written in terms of the fourvectors $U_{\mu }=p_{\mu }-B_{\mu }$ and $V_{\mu }=p_{\mu }+B_{\mu }$ as

B3 $$\begin{aligned} U_{\mu }U^{\mu }\;V_{\nu }V^{\nu }-2m^2 \; U_{\mu }V^{\mu }+m^4=0. \end{aligned}$$

Hence for a fixed spatial momentum $\mathbf {p}$ there are four different values of the energy. Due to C-invariance of the operator $\overline{\psi }\gamma ^5\gamma _\mu \psi$ the energy levels come in pairs with opposite sign. The relations found are the same as one would find when looking for the poles of the fermion propagator in [44, 69].

At this point we would like to observe that the dispersion relations in the paper [70–73] $(p_a\pm B_a)=m^2$ (written in the tetrad frame in curved space, but supposedly valid also in flat space if one allows for torsion) are in general *not* compatible with (B3) for massive fermions. In particular they are not in the case $\mathbf {B}=0$, $B_0\ne 0$. The peculiar form of (B3) is essentially due to the chiral nature of the coupling. Because of the very definition of a fermion mass term, there is neither a natural way of splitting the two chiral components in the massive case nor identifying them with particles and antiparticles. Also it is not clear under what conditions the field $B_a$ can be constant throughout on a curved space-time, and even how plane-wave solutions found in the non-holonomic basis. For a further discussion of this subject see [74], where the incorrectness of [70–73] is shown to follow from the non-tensorial transformation properties of the pseudovector $B_a$. The latter is a peculiar property of curved space-times, since on flat space-times the only contribution to the connection comes from the torsion tensor. As such, the strong equivalence principle implies that, in Riemannian spaces without torsion, locally one can always find a frame where the space-time is flat, thus eliminating $B_a$, which therefore cannot contain covariant information such as the one leading to leptogenesis. In contrast, the presence of a torsion field leads to a proper axial vector background coupled to fermions, which under certain circumstances may be constant in some frame, leading to ${ CP }$- and CPT-violating Leptogenesis, e.g. the case of stringy cosmologies [40], where there is a constant antisymmetric tensor field strength background in the Robertson–Walker frame.

From now on, unless otherwise specified, the constant field $B_{\mu }$ will be taken parallel to the time axis. This field value represents a vector vacuum expectation value that is responsible for the spontaneous breakdown of *particle Lorentz invariance* (as defined in [44]). A reason for this choice can be found in the papers [40, 43], where solutions for the Kalb–Ramond field in the expanding Universe are explicitly given. In fact, in that case $H_{\mu \nu \rho }=e^{2\phi }\varepsilon _{\mu \nu \rho \sigma }\partial ^{\sigma }b(x)$ and the axion field *b*(*x*) is linear in time, thus entailing a purely timelike $B_{\mu }$. The positive frequency spinors are given by

B4 $$\begin{aligned} u_r(p)&=\left( \begin{array}{l} \sqrt{E_r-B_0-\lambda _r|\vec {p}|} \;\xi ^r\\ \sqrt{E_r+B_0+\lambda _r|\vec {p}|} \;\xi ^r \end{array}\right) \nonumber \\ {}&=\left( \begin{array}{l} \sqrt{E_r-B_0-\vec {p}\cdot \vec {\sigma }}\;\xi ^r\\ \sqrt{E_r+B_0+\vec {p}\cdot \vec {\sigma }}\;\xi ^r \end{array}\right) . \end{aligned}$$

$\xi ^r(\vec {p})$ are the usual helicity eigenspinors

B5 $$\begin{aligned} \frac{\vec {p}\cdot \vec {\sigma }}{|\vec {p}|} \; \xi ^r = \lambda _r \; \xi ^r, \quad r=1,2 \end{aligned}$$

where $\lambda _r\equiv (-1)^{r-1}$. The spinors are taken to be orthonormal, i.e. $\xi ^{r\dagger }\xi ^{s}=\delta ^{rs}$. $E_r$ is the energy corresponding to $\lambda _r$ via the relation

B6 $$\begin{aligned} E_r^2=m^2+(B_0+\lambda _r |\mathbf {p}|)^2. \end{aligned}$$

The last equation is a particular case of (B3), corresponding to $\mathbf {B}=0$. This result agrees with the formulae in [44, 75].

On the other hand, *negative-frequency* solutions are given by

B7 $$\begin{aligned} v_s(p)=\left( \begin{array}{l} \sqrt{E_s+B_0-\vec {p}\cdot \vec {\sigma }} \;\xi ^s\\ -\sqrt{E_s-B_0+\vec {p}\cdot \vec {\sigma }} \;\xi ^s \end{array}\right) . \end{aligned}$$

Notice that here the corresponding dispersion relations appear to be inverted if compared to the previous case. In other words, $E_1$ corresponds to $s=2$ and $E_2$ to $s=1$. This is to be expected on the basis of Dirac’s hole theory.

The spinors satisfy the following normalisation conditions:

B8 $$\begin{aligned} \overline{u}_r u_s=2m\;\delta _{rs}, \quad \overline{v}_r v_s=-2m\;\delta _{rs}, \end{aligned}$$

or equivalently

B9 $$\begin{aligned} u_r^\dagger u_s=2E_r\;\delta _{rs}, \quad \overline{v}_r v_s=2E_r\;\delta _{rs}. \end{aligned}$$

It is important to stress that these solutions only hold in the frame where $B_{\mu }$ is purely temporal. Furthermore, as a consequence of broken *particle Lorentz invariance*, the spinor wave function of a particle with momentum $\mathbf {p}$ cannot be obtained by boosting the solution for a spinor at rest.

The Dirac field operator is a straightforward generalisation of the standard one and is constructed from the plane-wave solutions of the Dirac equation [76]

B10 $$\begin{aligned} \psi (x)= & {} \int \frac{\text{ d }^3p}{(2\pi )^3}\sum _{r=1,2}\frac{1}{\sqrt{2E_r}}\nonumber \\&\times \left( a^{r}_{\mathbf {p}}u^r(p)e^{-ipx}+b^{r\dagger }_{\mathbf {p}}v^r(p)e^{ipx}\right) . \end{aligned}$$

The only difference with the standard case is that here the energy depends on the helicity. As usual, canonical equal-time anti-commutation relations must be imposed on the fields and this leads in turn to the fermionic oscillator algebra of the creation and annihilation operators $a^{r}_{\mathbf {p}}$, $a^{r \; \dagger }_{\mathbf {p}}$ and $b^{r}_{\mathbf {p}}$, $b^{r \; \dagger }_{\mathbf {p}}$.

### Inequivalence of helicity and chirality in the presence of an axial background

It is well known that, in the massless limit, the action of the chirality and the helicity operator on plane-wave solutions of the standard Dirac equation is the same. One can then ask whether this basic result still holds in the CPT-violating theory considered. It turns out that the answer is negative, as it is readily seen with a simple example.

Let us consider a positive frequency, positive helicity spinor, as given by Eq. (B4), with $m=0$

B11 $$\begin{aligned} u_{1}=\left( \begin{array}{l} \sqrt{E_{1}-B_0-|\vec {p}|}\xi _{1}\\ \sqrt{E_{1}+B_0+|\vec {p}|} \xi _{1} \end{array}\right) . \end{aligned}$$

We have from (B6)

B12 $$\begin{aligned} E_{1}=\left|B_0+|\vec {p}|\right|. \end{aligned}$$

When $B_0$ is non-zero, this leads to two different cases, depending on the sign of the argument of the absolute value function. Therefore, if $B_0+|\vec {p}|\ge 0$ the spinor has right chirality, while $B_0+|\vec {p}|<0$ implies that it has left chirality! Obviously when $B_0=0$ the second case is forbidden, hence re-establishing the usual correspondence.

## Appendix C: Kinematics of the decay of the right-handed neutrino to first order in the external field

In this appendix we illustrate how the kinematics of the right-handed Majorana neutrino decay processes considered in Sect. 2 can be studied using simple perturbative techniques. We shall consider the case $N\longrightarrow l^- \overline{\phi }$ for definiteness. The other decay mode can be studied in an analogous way and will not be presented here. All we need to know is that the total energy and the total momentum are both conserved in the process

C1 $$\begin{aligned} E_{N,r}&=E_{l^-,s}+E_{\overline{\phi }},\end{aligned}$$

C2 $$\begin{aligned} \mathbf {p}&=\mathbf {q}+\mathbf {k}. \end{aligned}$$

Lorentz violation, due to the external background field $B_0$ (4), introduces corrections in the first equation through the dependence of the energies of the decaying neutrino and of the lepton on the helicity and on the background field.

C3 $$\begin{aligned} E_{N,r}&=\sqrt{m^2 +(p+\lambda _{r} B_0)^2}\end{aligned}$$

C4 $$\begin{aligned} E_{l^-,s}&=|B_0+\lambda _s q|=-(B_0+\lambda _s q) \end{aligned}$$

Here *p* and *q* represent, respectively, the norms of the spatial vectors $\mathbf {p}$ and $\mathbf {q}$. The final step in the last equation follows from (16), where we showed that only if the argument of the absolute value is negative can the amplitude be different from zero. In principle, one can solve this system of equations for *q*, *k* and $\theta _{pq}$ (the angle formed by the vectors $\mathbf {p}$ and $\mathbf {q}$) in terms of *p*, of the helicities, the external field $B_0$ and the angle $\theta _{pk}$ (formed by $\mathbf {p}$ and $\mathbf {k}$). However, as it stands, it is hard to find a solution for the system of equations (C3).

It is convenient to treat the corrections coming from Lorentz violation as perturbations (which is justified if $B_0\ll m$, *T*) and define the solutions of the system as the sum of the unperturbed ones plus perturbations,

C5 $$\begin{aligned}&k=k^0+k^1,\end{aligned}$$

C6 $$\begin{aligned}&q=q^0+q^1,\end{aligned}$$

C7 $$\begin{aligned}&\theta _{pq}=\theta _{pq}^0+\theta _{pq}^1. \end{aligned}$$

Introducing the adimensional parameter $\varepsilon =\frac{B_0}{m}$, that will play the role of an expansion parameter, we write down the energy of the Majorana neutrino to first order in $\varepsilon$

C8 $$\begin{aligned} E_{N,r}=E_{N}^0\left( 1+\varepsilon \lambda _{r} \alpha \frac{p}{m}\right) +\mathcal {O}(\varepsilon ^2). \end{aligned}$$

Having defined $\alpha =\frac{m^{2}}{(E_{N}^0)^2}$. We get the linearised system of equations

C9 $$\begin{aligned}&k^1-\lambda _{s} \;q^1= C,\end{aligned}$$

C10 $$\begin{aligned}&\sin \theta _{pk}\; k^1 +\sin \theta _{pq}^0 \;q^1+q^0\cos \theta _{pq}^0 \;\theta _{pq}^1=0,\end{aligned}$$

C11 $$\begin{aligned}&\cos \theta _{pk} \;k^1 +\cos \theta _{pq}^0 \;q^1 - q^0 \sin \theta _{pq}^0 \;\theta _{pq}^1=0, \end{aligned}$$

which has the following solution:

C12 $$\begin{aligned} k^1&=\frac{C}{1+\lambda _s\cos \big (\theta _{pk}-\theta _{pq}^0\big )},\end{aligned}$$

C13 $$\begin{aligned} q^1&=-\frac{C \cos \big (\theta _{pk}-\theta _{pq}^0\big )}{1+\lambda _s \cos \big (\theta _{pk}-\theta _{pq}^0\big )},\end{aligned}$$

C14 $$\begin{aligned} \theta _{pq}^1&=-\frac{C\sin \big (\theta _{pk}-\theta _{pq}^0\big )}{q^0\big (1+\lambda _s\cos \big (\theta _{pk}-\theta _{pq}^0\big )\big )}. \end{aligned}$$

The definition of *C* is

C15 $$\begin{aligned} C=\varepsilon \lambda _{r} \alpha \frac{p}{m} E_{N}^0+(1+\lambda _s)q^0+B_0. \end{aligned}$$

In the centre of mass frame $\theta _{pk}=\theta _{pq}^0 + \pi$, implying that only $\lambda =-1$ is allowed and the usual correspondence between helicity and chirality is re-established. This is in agreement with Eq. (16), which was derived independently.

## Appendix D: Towards a more quantitative estimate of lepton abundance: Boltzmann equation in the presence of $B_0 \ne 0$ axial background

The Boltzmann equation [47] essentially expresses the action of the Liouville operator $\widehat{L}[f]$ on the phase-space density of the species $\chi$, $f({\vec {x}}, |{\vec {p}}|, t )$, in terms of the so-called collision operator *C*[*f*], monitoring the deviation from equilibrium in the reactions that the species $\chi$ participates (for an application of the Boltzmann equation to simple model of baryogenesis see e.g. [77]). We assume for concreteness one single dominant species, with mass $m_\chi$.

In the non-relativistic (Newtonian) case, the Liouville operator is a total time derivative, time is universal, and ${\vec {x}} (t)$, ${\vec {p}}(t)$ depend on time (phase-space trajectory of the particle): so its action on $f({\vec {x}}, |{\vec {p}}|, t )$ is given by

D1 $$\begin{aligned} {\widehat{L}}[f] = \frac{d}{\mathrm{d}t} f = \frac{\partial }{\partial t} f + {\vec {v}}\cdot {\vec {\nabla }} f + \frac{\vec {F}}{m_\chi }\cdot \nabla _{\vec {v}} f \end{aligned}$$

where $\vec {v} = \mathrm{d}\vec x/\mathrm{d}t$ is the velocity, and $\vec {F} = d\vec {p}/\mathrm{d}t$ is the (Newtonian) force acting on the particle.

The extension of (D1) to the general-relativistic case that will allow treatment in the Robertson–Walker Universe is straightforward. Essentially, the Newtonian total time derivative of the non-relativistic case is replaced by a total *derivative* with respect to the *proper time*. The resulting Liouville operator is essentially,

D2 $$\begin{aligned} \widehat{L}[f] \rightarrow m_\chi \frac{d}{\mathrm{d}\tau } f = m_\chi u^\alpha \partial _\alpha f + m_\chi \frac{\mathrm{d} p^\alpha }{\mathrm{d} \tau } \frac{\partial }{\partial p^\alpha } f, \end{aligned}$$

where $u^\mu$ is the four-velocity and $p^\mu = m_\chi u^\mu$ the four-momentum. In (D2) we took into account that any dependence of the phase-space density *f* on the proper time $\tau$ is through the dependence of $x^\mu (\tau ), p^\alpha (\tau )$ on $\tau$.

Based on our discussion so far, then, the combination $\frac{\partial }{\partial t} f + {\vec v}\cdot {\vec \nabla }$ of the Newtonian case is replaced in general relativity by $u^\alpha \partial _\alpha$, whilst the ‘force’ term is expressed in terms of the Christoffel symbols by means of the geodesic equation,

D3 $$\begin{aligned} m_\chi \frac{\mathrm{d} p^\alpha }{\mathrm{d} \tau } = - \Gamma ^\mu _{\alpha \beta } p^\alpha p^\beta . \end{aligned}$$

Notice that the torsion (antisymmetric part of the Christoffel symbol) does not enter the geodesic equation. Nevertheless, as we shall discuss below, the equation is still modified by the presence of the $B_0$ vector, due to the modified dispersion relations (B6) for the various helicity states.

The result for the general-relativistic Liouville operator is, therefore,

D4 $$\begin{aligned} \widehat{L}[f] = \left[ p^\alpha \partial _\alpha - \Gamma ^\alpha _{\mu \nu }p^\mu p^\nu \frac{\partial }{\partial p^\alpha } \right] f. \end{aligned}$$

For a homogeneous and isotropic Robertson–Walker Universe, with a scale factor *a*(*t*), we have $f=f(t,|\vec p|)$. Equivalently, upon using the RW-space-time on-shell condition for the massive species $\chi$, $f=f(E,t)$, where *E* denotes the energy of the particle and *t* is the co-moving frame RW cosmic time. On using the Christoffel symbols for the Robertson–Walker metric, we obtain from (D4):

D5 $$\begin{aligned} \widehat{L}[f] = E \frac{\partial f}{\partial t} - \frac{\dot{a}}{a}|\vec p|^2 \frac{\partial f}{\partial E}. \end{aligned}$$

The number density of species $n_\chi$ is defined as:

D6 $$\begin{aligned} n_\chi = \frac{g}{8\pi ^3}\int \mathrm{d}^3p f(E,t) \end{aligned}$$

where *g* is the number of degrees of freedom of the species $\chi$. Dividing (D5) by *E*, integrating over all momenta and using the definition (D6), we obtain

D7 $$\begin{aligned} \frac{\mathrm{d} n_\chi }{\mathrm{d} t} - \frac{\dot{a}}{a} \frac{g}{8\pi ^3}\int _{0}^{\infty } \mathrm{d}|\vec p| \mathrm{d}\Omega ~ \frac{|\vec p|^4}{E} \frac{\partial |\vec p|}{\partial E} \frac{\partial f}{\partial |\vec p|} \end{aligned}$$

where in the last step we have spilt the momentum integration into momentum-amplitude ($|\vec p|$) and angular ($\Omega$) parts, and transformed the *E*-differentiation to a $|\vec p|$-differentiation. Consider now the abundance of a particular helicity state $\chi = r$, $n_r$. Using the dispersion relations (B6) we obtain

$$\begin{aligned} \frac{\partial |\vec p|}{\partial E_r} = \frac{E_r}{|\vec p| + \lambda _r \, B_0}, \quad \lambda _r = \pm 1. \end{aligned}$$

with the notation $|\vec p|^2 \equiv p_i p_j h^{ij}$, where $h_{ij}$ is the spatial part of the RW metric in the standard notation. For concreteess and consistency with astrophysical observations (if one neglects the small value of the cosmological constant), we may assume that the Universe is spatially flat, in which case $h_{ij}=\delta _{ij}$.

Notice that, depending on the sign of $B_0$, the quantity $|\vec p | + \lambda _r B_0$ may vanish. However, the integrand of (D7) is regular, as $\partial f /\partial |\vec p| \propto |\vec p| + \lambda _r B_0$, for the Boltzmann (thermal) distribution at temperature *T*

$$\begin{aligned} f(E_r; T) = \frac{1}{e^{E_r/T} + 1}, \end{aligned}$$

assuming zero chemical potential for the relativistic right-handed neutrinos of helicity *r* for simplicity, with $E_r$ given by (B6). With this in mind, we can expand (D7) in powers of $B_0$ (small compated to the temperature $T_D$) and integrate by parts to arrive at a modified Boltzmann equation for the number density of a helicity state *r* in the form

D8 $$\begin{aligned}&\frac{g}{8\pi ^3}\int \frac{\mathrm{d}^3p}{E}C[f] = \mathrm{d}n_r/\mathrm{d}t + \frac{g}{8\pi ^3} \, \frac{{\dot{a}}}{a}\nonumber \\&\qquad \times \int \, \mathrm{d}|\vec p| \mathrm{d}\Omega ~ \frac{\partial }{\partial |\vec p|} \left( \frac{|\vec p|^4}{|\vec p| + \lambda _r \, B_0}\right) \, f \nonumber \\&\quad \simeq \mathrm{d}n_r/\mathrm{d}t + 3Hn_r - \frac{g}{2\pi ^2} \, 2\lambda _r \, B_0 \int \mathrm{d}|\vec p| \, |\vec p| \, f + {\mathcal O}(B_0^2), \end{aligned}$$

where $H = \frac{\dot{a}}{a}$ is the Hubble parameter of the Universe. If we restrict ourselves to small $B_0/T \ll 1$, which is to be expected from our qualitative estimates in the previous subsection, then we may ignore any $B_0$ dependence of *f* in the last integral of the right-hand-side of (D8), and thus replace *f* by the standard Boltzmann distribution of a particle of mass *m* with energy $E(B_0) = \sqrt{m^2 + |\vec p|^2}$. On defining the dimensionless variable $|\vec {p}|/T \equiv u$, we obtain the modified Boltzmann

D9 $$\begin{aligned}&\mathrm{d}n_r/\mathrm{d}t + 3Hn_r - \frac{g}{2\pi ^2} \, 2\lambda _r \, \frac{B_0}{T}\, T^3 \int \mathrm{d}u \, u \, f (E(B_0=0), u) \nonumber \\&\quad =\frac{g}{8\pi ^3}\int \frac{d^3p}{E(B_0 \ne 0)}C[f] + {\mathcal O}(B_0^2) \end{aligned}$$

where on the right-hand side the $B_0$ dependent energy is used.

Equation (D9) holds for any given species, with a suitable collision operator. In particular, for the right-handed neutrino, we may sum the contributions coming from opposite helicities and get an equation for the total density.

D10 $$\begin{aligned} \frac{\text{ d } n_{N}}{\text{ d }t}+3Hn_{N}=\frac{g}{8\pi ^3}\int \frac{\mathrm{d}^3p}{E}\tilde{C}[f] + {\mathcal O}(B_0^2) \end{aligned}$$

The collision operator must include contributions for the direct and inverse decays, as well as for the processes with $\Delta L= 1, 2$ considered in [8, 37].

## Footnotes

1 A pedagogical discussion of the necessity (in the absence of torsion) of interference between tree-level and one-loop diagrams can be found in [5].

2 In these scenarios the quantum fluctuations of the four-dimensional torsion arising from string theory give rise to a physical KR axion-like field. This field couples, with the divergence of the axial fermion currents (non-zero due to the chiral anomalies), as well as with ordinary axion fields, assumed in the spectrum, via the respective kinetic terms. Standard axions couple, via appropriate chirality changing Yukawa interactions, to massless chiral neutrino fields. Without the need for any specific number of right-handed neutrinos, such Yukawa couplings generate Majorana masses for the chiral neutrino fields of the SM sector through higher-loop anomalous graphs involving graviton fields, We could then embed our mechanism for leptogenesis into such a scenario, with just one right-handed neutrino but with the assumption that masses for all the left-handed neutrinos are generated via this new mechanism.

3 For an alternative derivation, not involving Erf functions, one can consider the approximate expression

$$\begin{aligned} I_{n}(a,\beta ,m)\simeq \int _{0}^{\infty }\mathrm{d}p (p+a)^{n}f(p)\exp (-\beta \, p) \end{aligned}$$

where $$f(p)=e^{-\beta \sqrt{a^{2}+m^{2}}} \left[ 1+p\left( \beta -\frac{\alpha \beta }{\sqrt{\alpha ^{2}+m^{2}}}\right) +\frac{1}{2}p^{2}\left( \left( \beta -\frac{\alpha \beta }{\sqrt{\alpha ^{2}+m^{2}}}\right) ^{2}-\frac{\beta m^{2}}{\left( \alpha ^{2}+m^{2}\right) ^{3/2}}\right) \right] $$.

4 At the level of the effective action (53), it can be seen that the *H*-field is non-propagating. As a consequence, in the path-integral approach to quantum theory, we can integrate out the antisymmetric torsion field strength $H_{\mu \nu \rho }$. However, in the context of a full string-inspired low-energy theory effective action, integrating out the torsion *H*-field is non-trivial: the action contains an infinity of higher-derivative interactions, for example those containing $(\nabla H)^2$ terms [59–61], that make the *H*-field a fully fledged propagating field with complicated interactions, of infinite order in $\alpha ^\prime$ (which are not known in closed form). Nevertheless, for our purposes here we shall assume weakly varying *H*-fields and hence one can restrict oneself to the lowest-order effective action (53) where the *H*-torsion can be integrated out, as a non-propagating field, mirroring the case of ordinary torsion in Einstein–Cartan theory, reviewed in Appendix A.
